# Chemical Synthesis of Human Proteoforms and Application in Biomedicine

**DOI:** 10.1021/acscentsci.4c00642

**Published:** 2024-07-22

**Authors:** Huasong Ai, Man Pan, Lei Liu

**Affiliations:** † New Cornerstone Science Laboratory, Tsinghua-Peking Joint Center for Life Sciences, MOE Key Laboratory of Bioorganic Phosphorus Chemistry and Chemical Biology, Center for Synthetic and Systems Biology, Department of Chemistry, Tsinghua University, Beijing 100084, China; ‡ Institute of Translational Medicine, School of Pharmacy, School of Chemistry and Chemical Engineering, National Center for Translational Medicine (Shanghai), Shanghai Jiao Tong University, Shanghai 200240, China

## Abstract

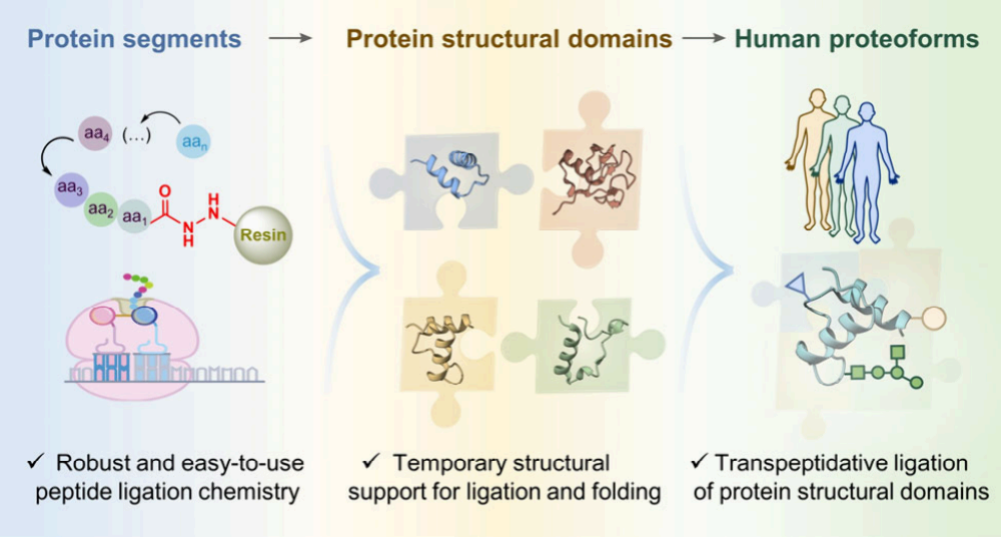

Limited understanding of human proteoforms with complex posttranslational modifications and the underlying mechanisms poses a major obstacle to research on human health and disease. This Outlook discusses opportunities and challenges of *de novo* chemical protein synthesis in human proteoform studies. Our analysis suggests that to develop a comprehensive, robust, and cost-effective methodology for chemical synthesis of various human proteoforms, new chemistries of the following types need to be developed: (1) easy-to-use peptide ligation chemistries allowing more efficient *de novo* synthesis of protein structural domains, (2) robust temporary structural support strategies for ligation and folding of challenging targets, and (3) efficient transpeptidative protein domain–domain ligation methods for multidomain proteins. Our analysis also indicates that accurate chemical synthesis of human proteoforms can be applied to the following aspects of biomedical research: (1) dissection and reconstitution of the proteoform interaction networks, (2) structural mechanism elucidation and functional analysis of human proteoform complexes, and (3) development and evaluation of drugs targeting human proteoforms. Overall, we suggest that through integrating chemical protein synthesis with *in vivo* functional analysis, mechanistic biochemistry, and drug development, synthetic chemistry would play a pivotal role in human proteoform research and facilitate the development of precision diagnostics and therapeutics.

## Abstract 2

The trilogy of chemical syntheses of the human proteoform offers a plausible approach to studying human proteoform biology and disease with precise resolution.

## Introduction

**Figure 1:**
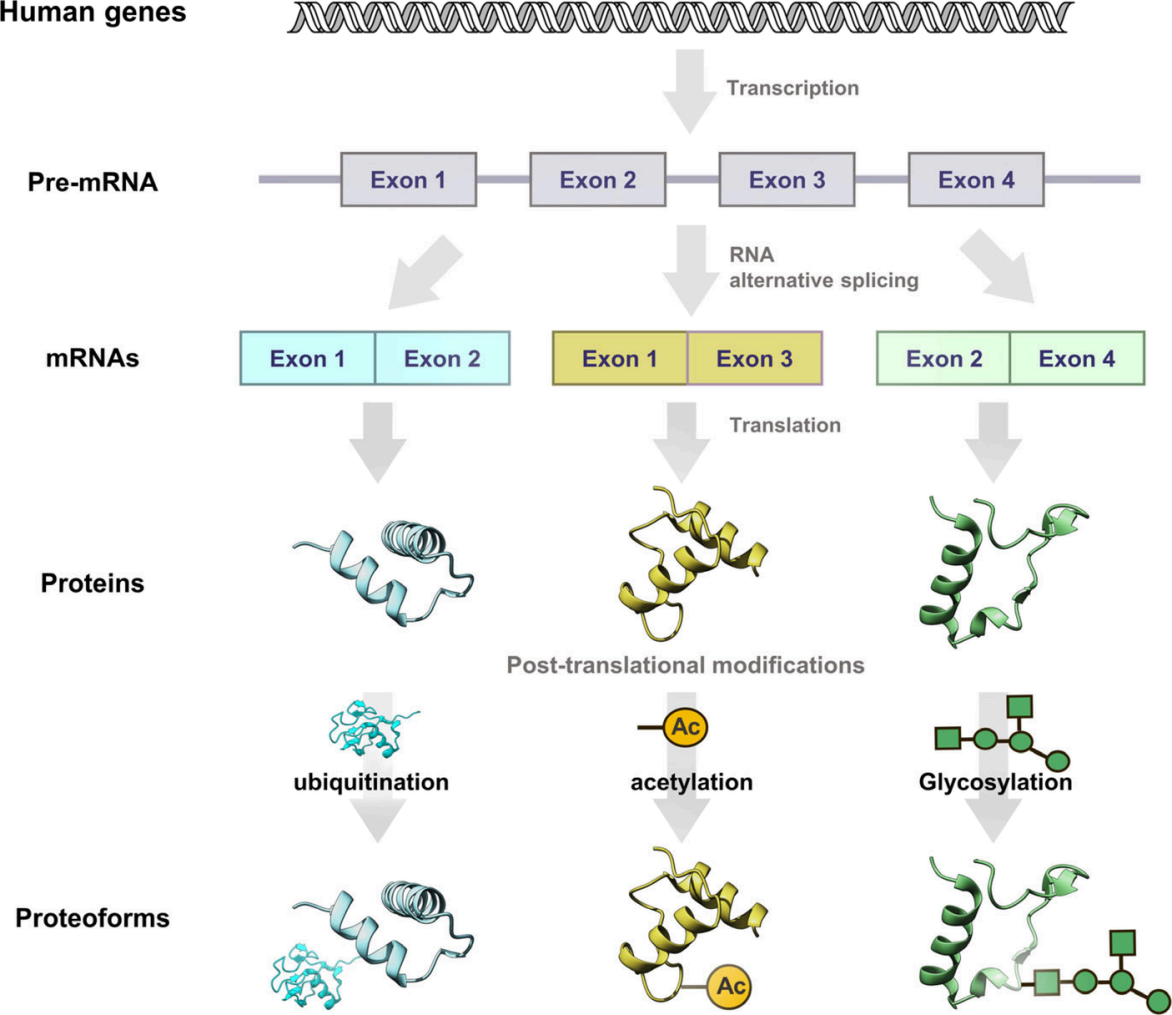
The proteoform paradigm. Human genes are transcribed into mRNAs via alternative splicing, and the resulting different spliced mature mRNAs are then translated into the indicated proteins, which undergo multiple PTMs (such as ubiquitination, acetylation, and glycosylation) to yield the final proteoforms.

Deciphering the human genetic code at the molecular level is a fundamental task in biomedical research. Although the “Human Genome Project” has successfully decoded the information carried by nucleic acids,^1,2^ our understanding of proteins, the direct executors of life activities and central intermediaries bridging genotype and phenotype, remains limited because the complexity of protein structures and functions extends well beyond the linear amino acid sequences determined by genetic codes. From the same genetic template, various factors including DNA genetic variation, RNA alternative splicing, and especially the diverse types and combinations of protein posttranslational modifications (PTMs) can lead to the generation of a wide range of structurally distinct proteins (Figure 1), known as proteoforms,^3^ which are the ultimate molecular effectors of biological function, posing challenges in inferring phenotypes based solely on genotypes. Preliminary estimations have indicated that human protein-encoding genes (approximately 20,000^4^) map to more than 10^7^ unique and nonredundant proteoforms,^5^ many of which are closely associated with diverse human diseases.^5,6^ Medical data from the past two decades have shown that genomic and transcriptomic variations can only adequately account for less than 10% of diseases that occur in clinical settings,^7−10^ underscoring the need to investigate numerous crucial targets omitted in protein-based diagnostic and therapeutic strategies.

**Figure 2:**
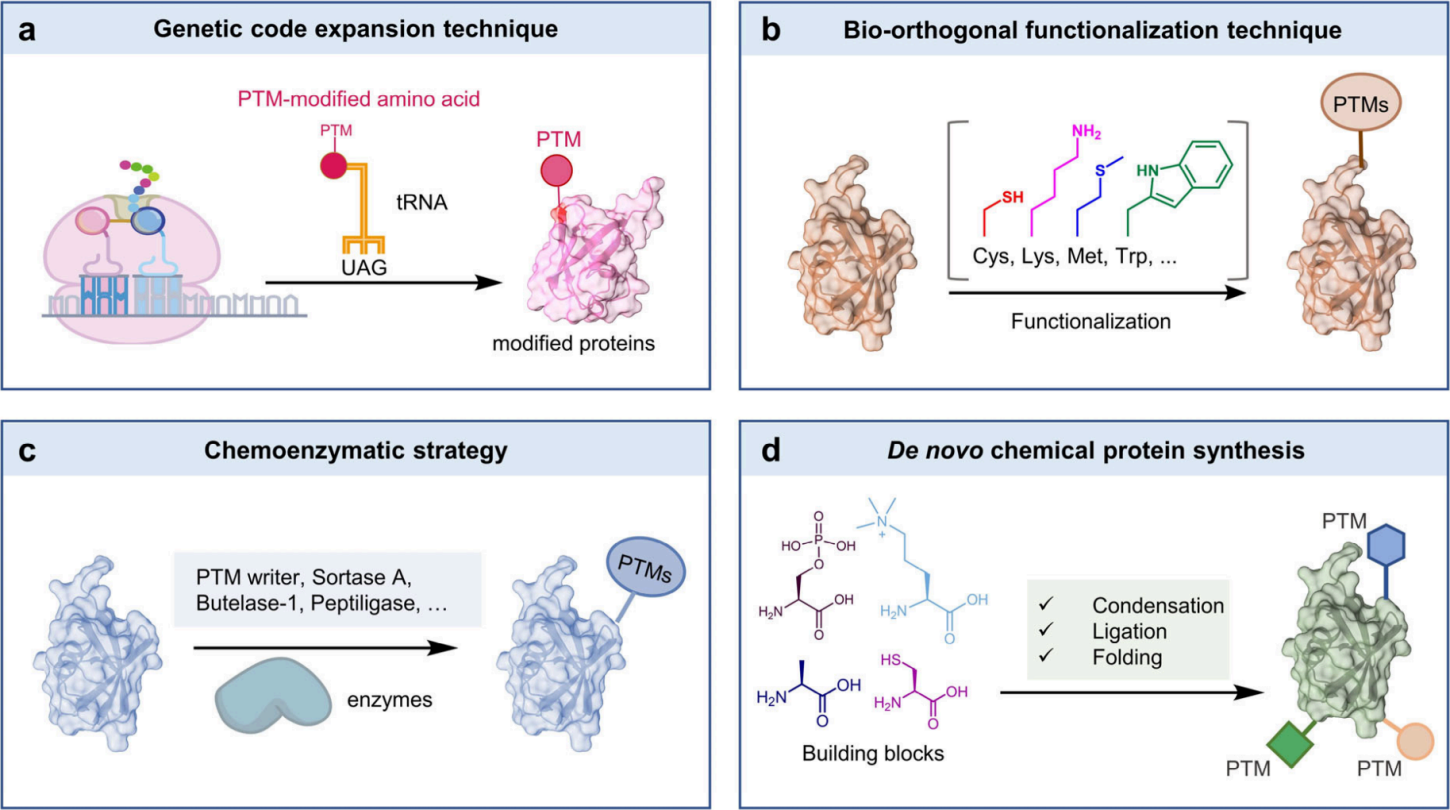
Synthesis of post-translationally modified proteins or proteoforms. Four current strategies can be used to prepare PTM proteins: genetic code expansion technique, bio-orthogonal functionalization technique, chemoenzymatic strategy, and *de novo* chemical protein synthesis.

**Figure 3:**
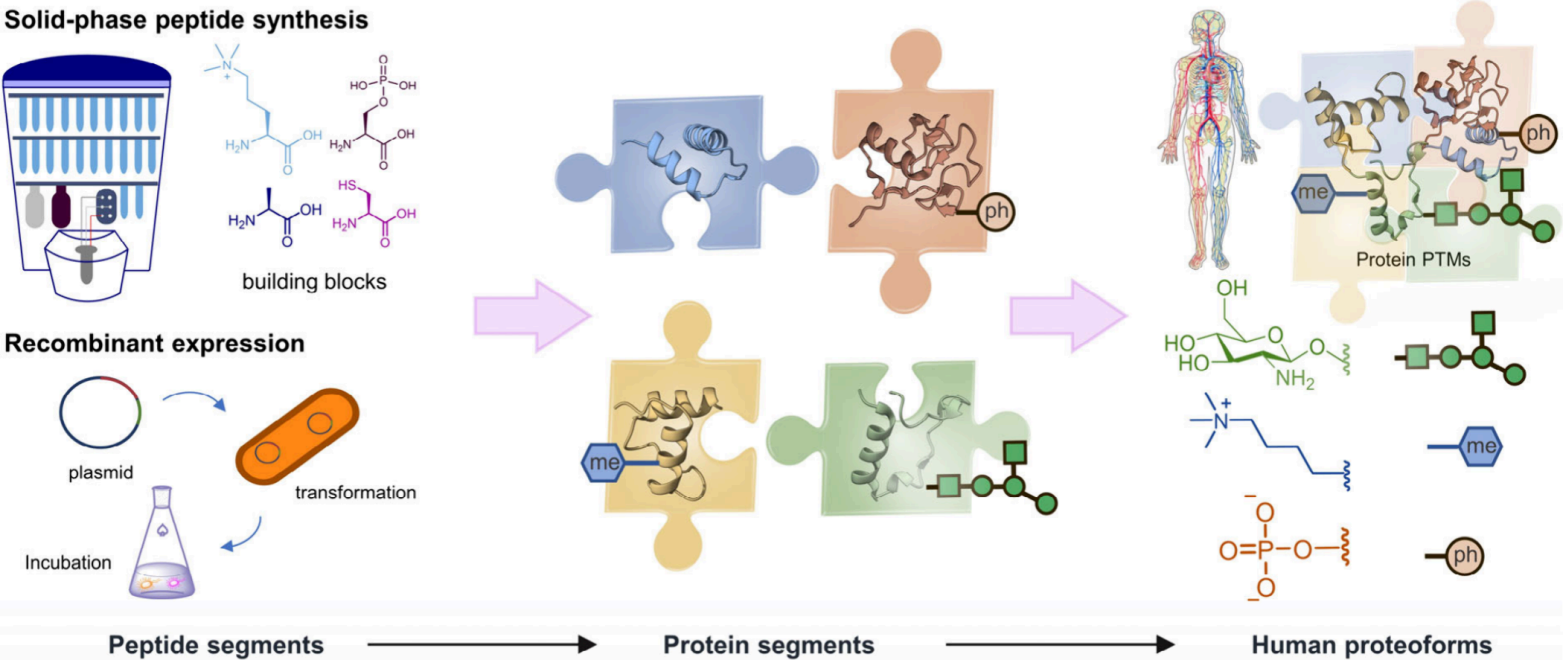
Conceptual diagram of chemical protein synthesis in human proteoforms. Protein segments bearing various PTMs, obtained from solid-phase peptide synthesis or recombinant expression, were assembled into protein structural domains followed by protein domain–domain ligation to obtain human proteoforms.

Uncovering the diversity and function of proteoforms is essential to biomedical research, including the dissection and analysis of the distributions and interactions of various proteoforms, systematic quantitative reconstitution of their biochemical events, and accurate elucidation of their molecular mechanisms at atomic resolution. However, revealing the causal relationships between the biological diversity of human proteoforms and the functional regulation of physiological and pathological processes has encountered challenges with traditional biological methods such as genetic recombinant expression or cellular extraction, which often struggle to provide structurally precise proteoform samples with defined PTMs required to support the research. In this context, chemical strategies have been developed to produce proteoforms with site-specific modifications First, genetic code expansion techniques enable the ribosomal integration of unnatural amino acids into proteins within living cells. The unnatural amino acids can carry modifications such as acylation and phosphorylation,^11−13^ while more complex PTMs (e.g., glycosylation, ubiquitination) remain difficult to incorporate due to challenge in the engineering of ribosomal aminoacyl-tRNA synthetases (Figure 2a). Second, bio-orthogonal functionalization techniques enable the preparation of site-selectively modified proteins at specific residues such as Cys.^14−19^ Lys,^20−22^ Met,^23−28^ and Trp^29−32^ of expressed protein (Figure 2b). PTM mimics are usually produced through this technology, which, however, may occasionally result in unforeseen biological consequences or ambiguity in interpreting experimental findings.^33^ Third, chemoenzymatic methods^34−36^ can be employed for obtaining proteoform samples where PTM enzymes (e.g., E3 ligase^37,38^) or other engineered enzymes (e.g., Sortase A,^39−41^ Butelase-1^42^) enable protein modification with site-selectivity (Figure 2c). This approach falls short for modifications lacking known enzymes or when the existing enzymes do not have sufficient substrate specificity. Finally, *de novo* chemical protein synthesis, a bottom-up approach for the construction of proteins, principally allows non-natural amino acids to be incorporated into a protein at any positions, and in any numbers and combinations.^43−45^ This approach could be more difficult to carry out, but it provides an avenue to complement the other methods for generating proteoforms with precise structures at atomic resolution^43,46,47^ (Figure 2d, 3).

Recently, there have been a number of comprehensive reviews on the development of genetic code expansion, bio-orthogonal functionalization, and chemoenzymatic methods for the studies of proteoforms.^11,34,48−61^ In this Outlook we focus on the opportunities and challenges of using chemical protein synthesis in studies on human proteoforms. We aim to advance the understanding of the role of chemical protein synthesis in biomedical research, unlocking the full potential of this field and embarking on the comprehensive exploration of human proteoforms.

## Chemistry Needed for the Synthesis of Human Proteoforms

Human proteins come in a wide array of sizes, shapes, and forms and play crucial roles in various life processes. This has led to a large gap between the knowledge of human genetics and the wide range of wellness and disease phenotypes. According to the Swiss-Prot database, the total number of human proteins is 20,434, with an average length of 558 residues and a median length of 415 residues (Table 1). These proteins stem from the continuous and precise translation of long-chain polypeptides by ribosomes in human cells,^62,63^ ranging in size from less than 50 amino acids to several hundred for most proteins (Table 1). Their diverse sizes, types (e.g., globular domain proteins, intrinsically disordered proteins), and complex physicochemical properties (e.g., hydrophobic transmembrane proteins, positively charged nucleolar proteins), coupled with combinations of myriad PTMs (e.g., methylation, acetylation, phosphorylation, and ubiquitination), pose challenges in acquiring these proteins using conventional biological methods, such as recombinant expression. In the past years, our team has been trying to develop a comprehensive, robust, and cost-effective methodology for the chemical synthesis of various human proteoforms, and we have identified the need to develop new chemistries of the following types: 1) easy-to-use peptide ligation chemistries that allow more efficient *de novo* synthesis of any protein structural domain; 2) robust strategies for providing temporary structural support for the ligation and folding of more challenging protein targets; and 3) efficient transpeptidative protein domain−domain ligation methods for the synthesis of multidomain proteins.

**Table 1:**
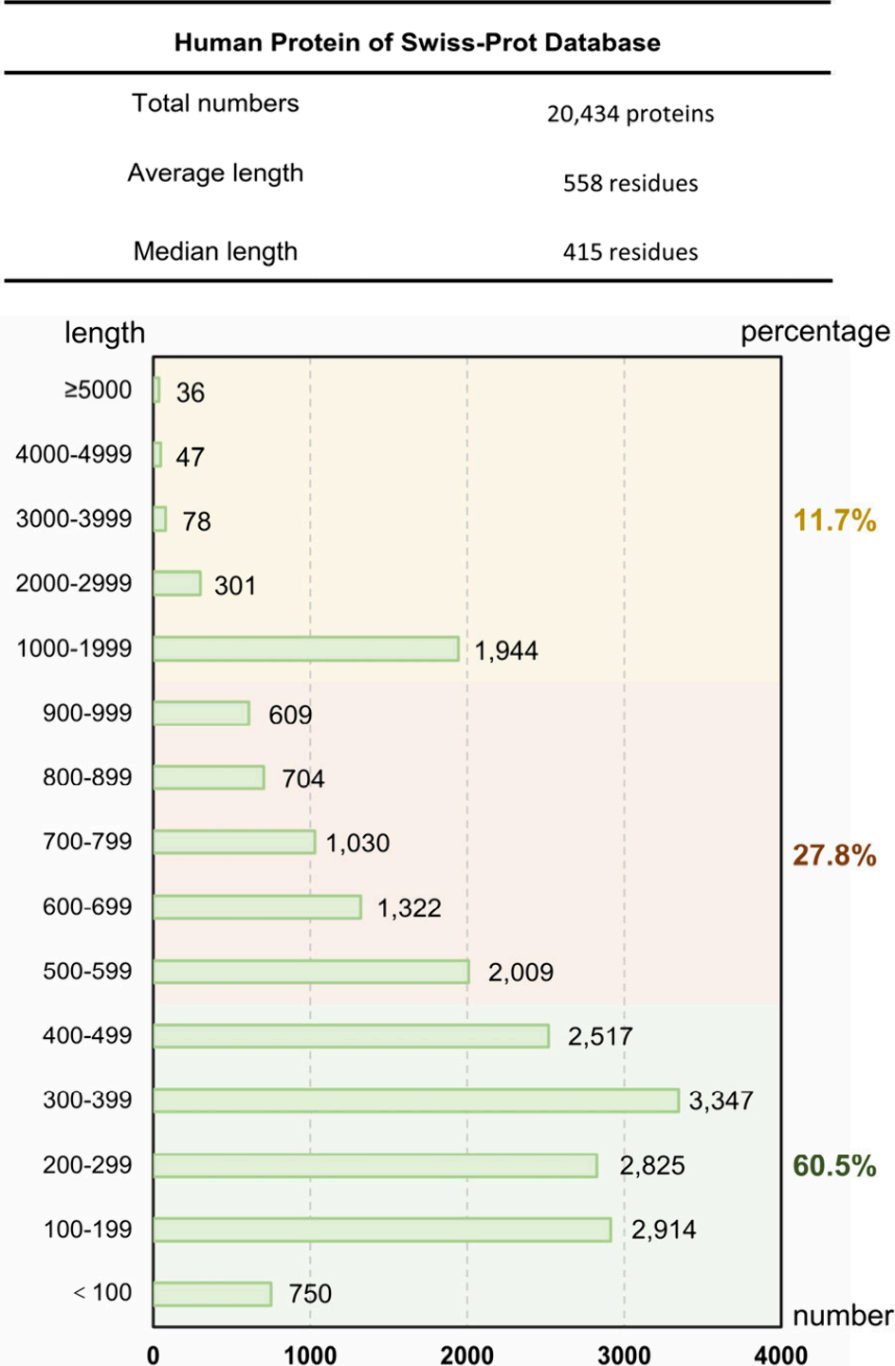
Protein Length Statistics of Human Proteomes

### Robust and Easy-to-Use Peptide Ligation Chemistry

Chemical protein synthesis has undergone substantial progress since the groundbreaking work of Merrifield and co-workers in solid-phase peptide synthesis (SPPS), a transformative technology^64^ that eliminates the need for intermediate separation after each amino acid coupling step, enabling the synthesis of peptide chains of usually up to ca. 50–100 amino acids that can be robustly purified.^59^ During SPPS, commercially available PTM-bearing amino acids (e.g., phosphorylated serine/threonine, mono/di/trimethylated or acetylated lysine) can be used, and polypeptides bearing site-specific PTMs can also be made. Furthermore, complex PTMs such as glycosylation and ubiquitination can be installed onto polypeptides through on-resin modification reactions or post-SPPS conjugation reactions. To achieve the synthesis of longer polypeptides in a cost-effective fashion, a transformative method known as native chemical ligation (NCL),^65^ which involves the use of a thiol-thioester exchange reaction between the thioester of one peptide segment and the Cys thiol group of another, was invented by Kent and co-workers in 1994. This powerful reaction produces a thioester-linked intermediate, which undergoes spontaneous intramolecular S, N-acyl transfer, resulting in the formation of a larger, purifiable peptide convergently from two short peptides.

**Figure 4:**
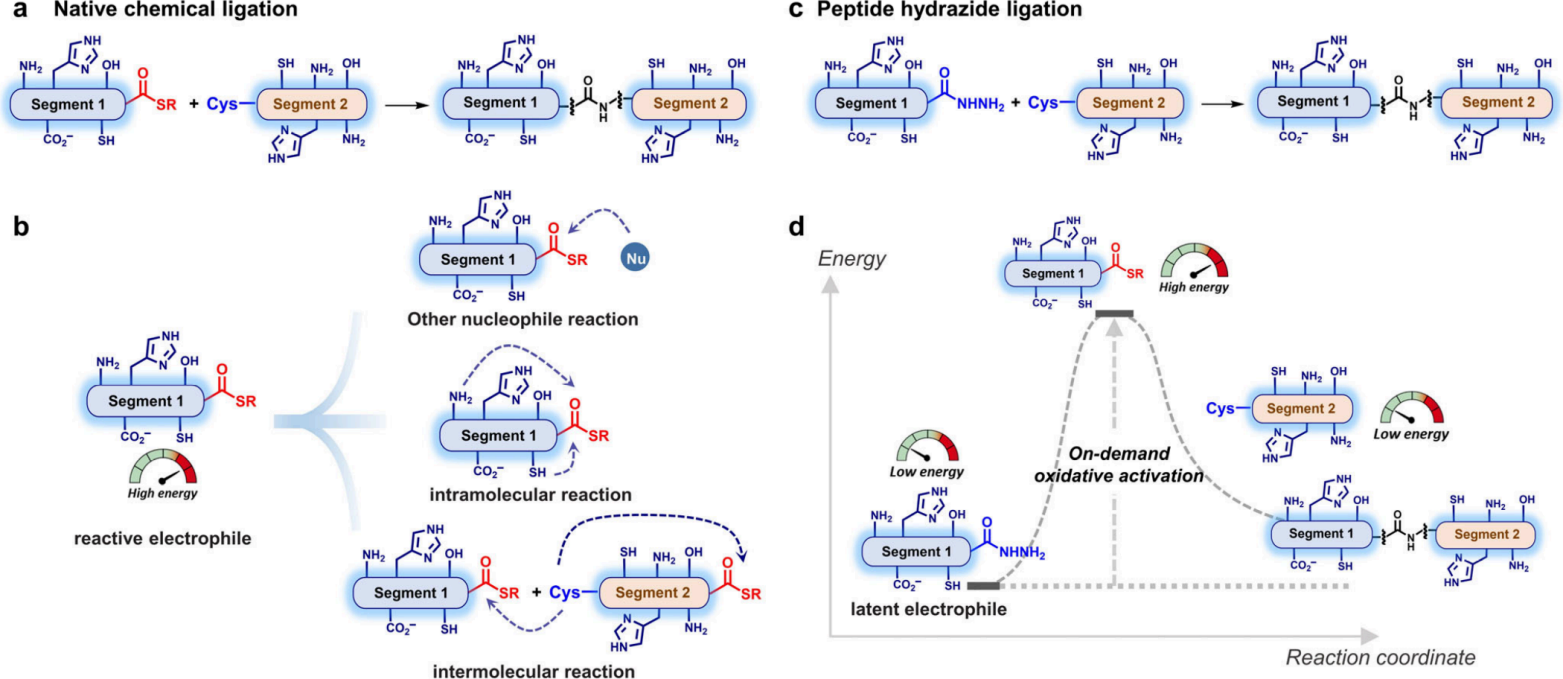
Chemical reactivity analysis of native chemical ligation and peptide hydrazide ligation. (a) Scheme of native chemical ligation of two peptides. (b) The shortcomings of high-energy electrophiles include instability caused by other nucleophilic reactions, intramolecular reactions, or intermolecular reactions. (c) Scheme of peptide hydrazide ligation. The low-energy peptide hydrazide was reacted with a Cys-containing peptide. (d) Energy illustration of the peptide hydrazide ligation intermediates. The low-energy peptide hydrazide (electrophile) was oxidatively activated on-demand to obtain the high-energy peptide thioester followed by ligation with low-energy Cys-containing peptides to obtain the assembled peptides.

The NCL approach has been widely used for chemical protein synthesis,^44,66−70^ but the robust acquisition of peptide thioesters that are sensitive to bases and nucleophiles has been a long-standing obstacle (Figure 4a-b). To overcome this hurdle, various approaches have been explored to develop peptide thioester surrogates that are compatible with Fmoc-SPPS conditions.^71−74^ Among them, peptide hydrazide ligation, which was discovered by us in 2011, has been recognized as one of the most efficient technologies for *de novo* chemical protein synthesis.^75^ This strategy employs redox chemistry to trigger on-demand chemoselective activation of otherwise inert peptide hydrazides by sodium nitrite (NaNO_2_) to generate reactive intermediates (acyl azide and thioester) in situ. These intermediates are capable of ligating with N-terminal Cys peptides (Figure 4c-d). Some important advantages of peptide hydrazide are the ease of large-scale preparation, stability in storage, and simple usage conditions, which greatly facilitate chemical protein synthesis.

Peptide hydrazides do not have any ligation reactivity until the hydrazide functional group is activated as needed. This makes peptide hydrazide ligation especially suitable for convergent protein synthesis requiring iterative ligations of multiple protein segments to obtain the target molecule^72^ (Figure 4b, 4d). A toolbox of hydrazide-based ligation strategies, including sequential hydrazide ligation,^76^ convergent hydrazide ligation and one-pot hydrazide ligation,^74^ has been developed. In addition to NaNO_2_, acetylacetone can also be used to activate peptide hydrazides.^77^ Due to the compatibility of the peptide hydrazide ligation with recombinantly accessed intein fusion proteins and protein segments generated through chemoenzymatic hydrazinolysis, an array of hydrazide-based protein semisynthesis methods have also been developed.^78,79^ Overall, the peptide hydrazide ligation provides an easy-to-use and robust approach for the chemical synthesis of a wide range of proteins including those bearing PTMs, protein probes, and mirror-image proteins, greatly broadening the range of targets accessible via chemical protein synthesis and semisynthesis.^80,81^ Proteins of up to ca. 200 amino acids can now be routinely prepared through the condensation of 2–4 peptide hydrazide segments, which cover 17.9% of the human proteome. For some highly soluble proteins, it is even feasible to ligate up to 10 peptide hydrazide segments, resulting in the synthesis of proteins bearing up to 500 amino acids.^47,82,83^

Note that the above ligation strategies were initially limited to reactions at Cys, while the development of thiolated amino acid-facilitated ligation followed by desulfurization has extended the range of available N-terminal residues^84−87^ due to the development of increasingly mild desulfurization strategies.^88−90^ Additionally, thiol-based auxiliaries, including trifluoroacetic acid-sensitive 1-aryl-2-mercaptoethyl auxiliary,^91,92^ photocleavable PEGylated auxiliary,^93,94^ and radical-cleavable 2-mercapto-2-(pyridin-2-yl)ethyl-(MPyE)-auxiliary,^95,96^ have also expanded the scope of ligation beyond Cys. Furthermore, the field of peptide ligation has been enriched by the development of other innovative technologies, including diselenide selenoester ligation,^97−100^ KAHA ligation,^101−103^ Staudinger ligation,^104−106^ Ser/Thr ligation^107,108^ and cysteine/penicillamine ligation.^109,110^ These strategies have advanced the synthesis of small to medium-sized human proteoforms, such as the core histone protein (H2A, H2B, H3 and H4 with residues ranging from 100–130) carrying complex types and combinations of PTMs,^111−115^ the nucleic acid-binding proteins like 160-residue transcription factor protein MAX/MYC with patterns of phosphorylation and acetylation,^116,117^ lipidated proteins like palmitoylated 178-residue caveolin-1,^118,119^ and glycoproteins like 312-residue ribonuclease B.^120−124^

### Temporary Structural Support for Ligation and Folding

**Figure 5:**
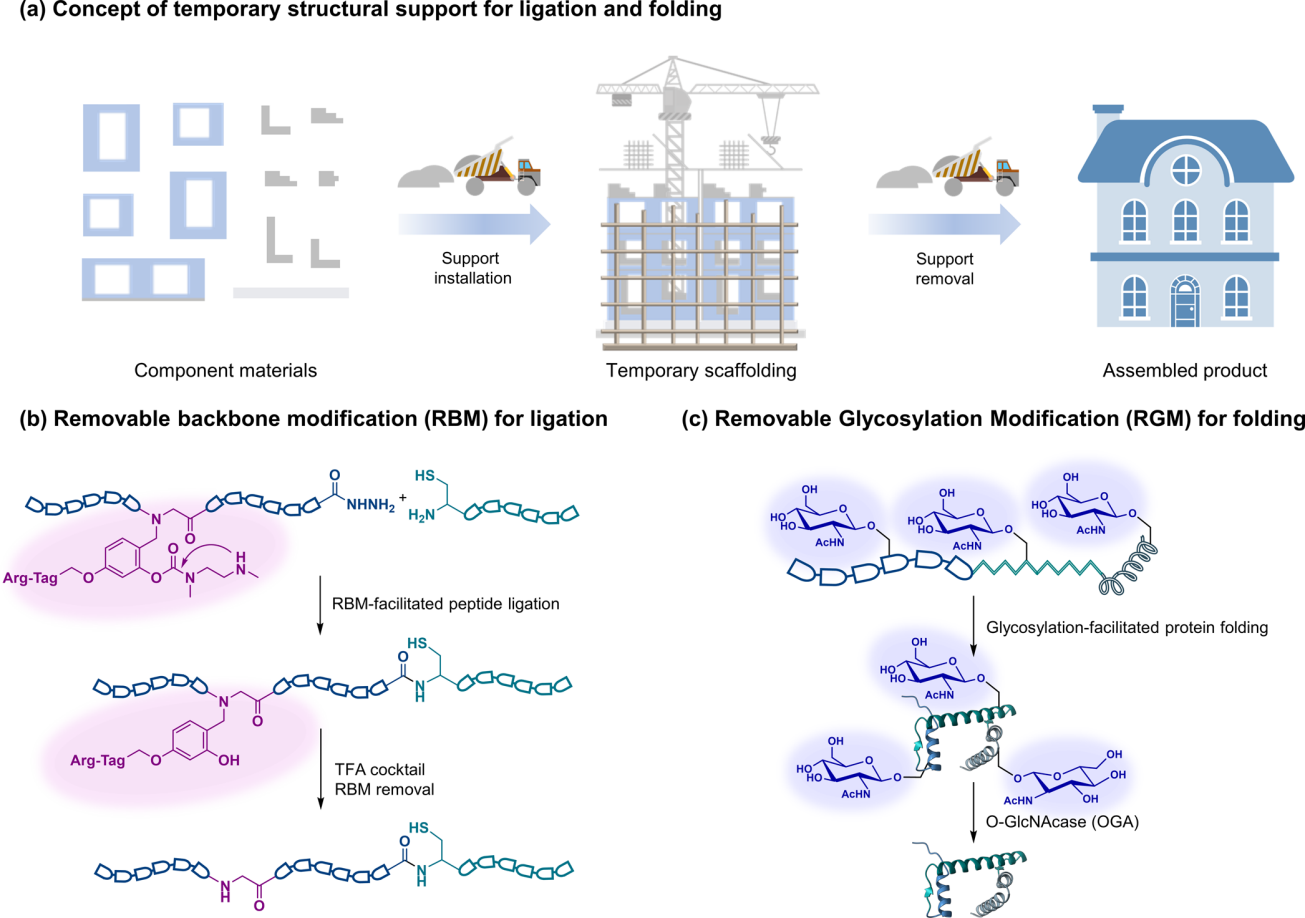
Temporary structural support for ligation and folding. (a) Concept scheme of the temporary structural support for building a house. (b) Representative case strategy of removable backbone modification (RBM) for peptide ligation. (c) Representative case strategy of removable glycosylation modification (RGM) for folding peptides into native proteins.

In the continuing studies on chemical protein synthesis, a series of increasingly challenging protein targets have been recognized that exhibit various unfavorable properties such as poor solubility, propensity to aggregate, and resistance to folding,^125−128^ for which synthesis has become very challenging or even impractical. To overcome this problem, we and others explored the concept of temporary structural support to intentionally manipulate the structures and properties of synthetic intermediates during protein ligation and folding. The basic idea behind the temporary support strategy can be compared to building a house or bridge in the macroscopic world, where temporary structural scaffolding is often strategically used to ensure the stability of the building at intermediate stages before the entire structure is completed (Figure 5a). By mimicking this idea and leveraging the remarkable tolerance of the peptide hydrazide ligation strategy to various chemical functionalities, we have developed several families of customized removable auxiliary groups as temporary structural supports and installed them onto synthetic protein intermediates to improve their ligation and folding behaviors.^128−130^

First, to overcome the challenge posed by the poor solubility of hydrophobic protein segments, we developed a removable backbone modification strategy to temporarily disrupt the hydrogen bonding between protein backbone amides, thereby blocking the formation of kinetically unfavorable secondary structures during protein synthesis (Figure 5b).^131,74,132^ This strategy has enabled the efficient chemical synthesis of transmembrane proteins, including the potassium ion channel Kir5.1, phosphorylated or S-palmitoylated influenza A virus M2 proton channel protein, isotope-labeled cation-specific ion channel p7, and multidrug resistance transporter protein EmrE,^131−133^ all of which are otherwise very difficult to synthesize. Notably, the removable backbone modification strategy was further used to overcome the soluble but unreactive propensity of segments of the *Haemophilus influenzae* DNA ligase Hin-Lig during synthesis, which disrupts formation of the soluble colloids to enable the desired peptide ligation.^134^

The concept of temporary structural support has also proven to be instrumental in addressing another persistent challenge in chemical protein synthesis, that is, protein folding. Inspired by *in vivo* protein folding mechanisms, where cells occasionally attach saccharides to protein sequences to facilitate proper folding, we temporarily installed glycosylation modifications (O-GlcNAc) on synthetic proteins. The O-GlcNAc groups enhanced the solubility of the folding intermediates and inhibited their interactions with each other. Subsequently, the modifications were completely removed from the proteins using O-GlcNAc glycosidase (OGA) (Figure 5c). Using this strategy, we accomplished the total synthesis of correctly folded cytokine interleukin-5 (IL-5) bearing four pairs of disulfides.^129^ Additionally, a recent study demonstrated that modification with O-GlcNAc, which can be cleaved by l-glycosidase enzymes, facilitates the chemical synthesis of correctly folded d-proteins including mirror-image tumor necrosis factor alpha (D-TNFα) and mirror-image receptor-binding domain of the Omicron spike protein (D-RBD).^130^

The methods of temporary structural support to promote protein ligation and folding, along with other tools such as isoacyl dipeptides^135−137^ and removable solubilizing tags,^138−146^ have expanded the capabilities of chemical protein synthesis, providing effective approaches for synthesizing increasingly challenging and larger protein targets. These advancements make it more effective to synthesize midsized human proteoforms with lengths of 300–500 amino acids, including those with poor water solubility (e.g., membrane proteins).^147^

### Transpeptidative Ligation of Protein Structural Domains

The development of peptide ligation chemistry has made it feasible to synthesize proteins within the range of 500 amino acids,^148,149^ covering 60.5% of the human proteome (Table 1). Peptide ligation reactions involving small segments demonstrate favorable kinetics when conducted at millimolar concentrations. However, as the molecular weight of the ligating segments increases, the efficiency of peptide ligation reactions based on random molecular collisions diminishes, particularly when the molecular weight exceeds 50 kDa. One possible reason is the burying of the ligation sites within the protein’s interior, rendering them inaccessible for the ligation reaction. Additionally, the low reactant solubility of large unfolded protein segments poses challenges in achieving the necessary millimolar concentration required for activity.

**Figure 6:**
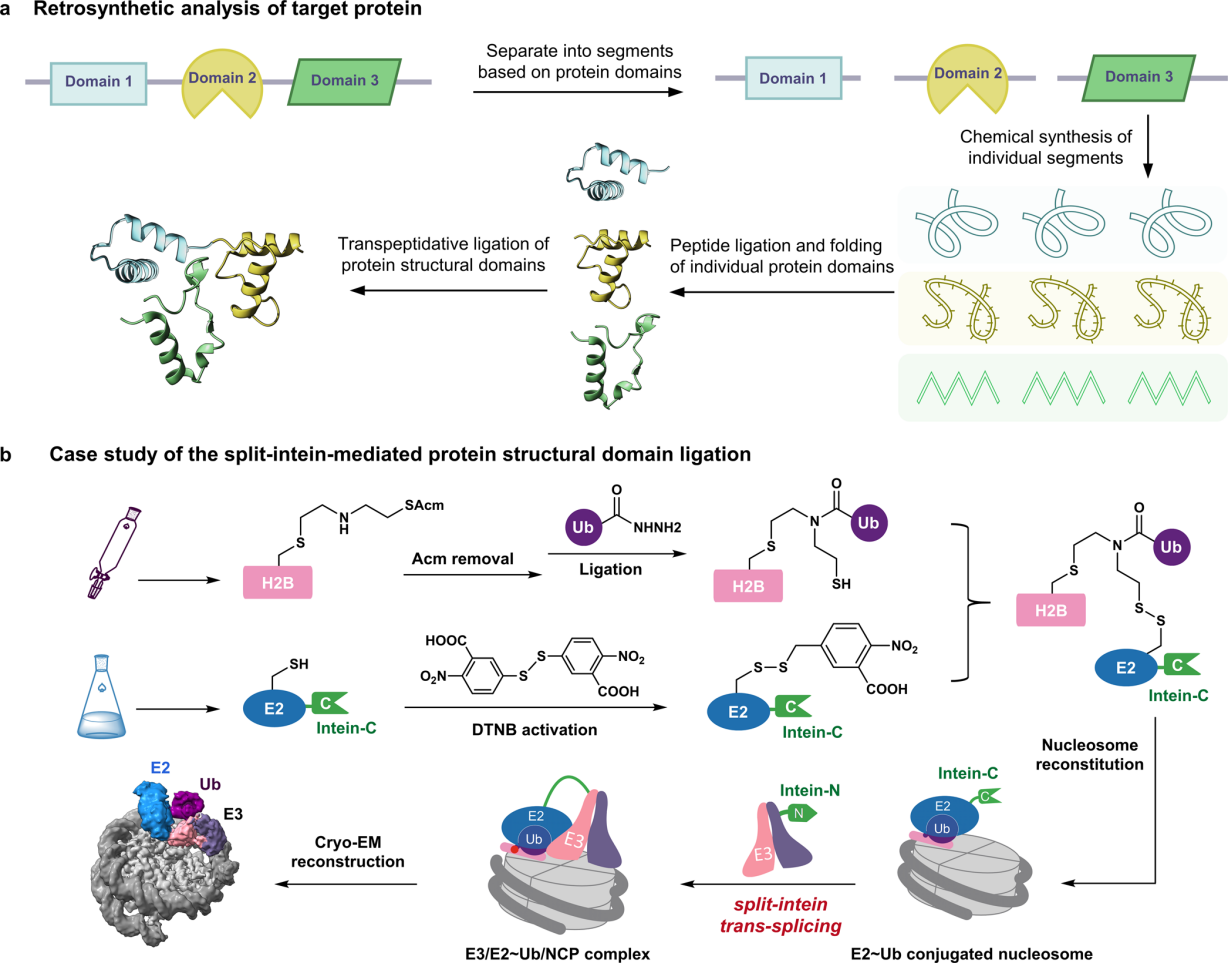
(a) Scheme for retrosynthetic analysis of a target protein. The protein was first segmented on the basis of protein domains followed by chemical synthesis of the peptides for each protein domain, peptide ligation and folding of individual protein domains, and final transpeptidative ligation of these protein structural domains. (b) Case study of the split-intein-mediated protein structural domain ligation of E3 and E2 enzymes for structural determination. Chemical synthesis of H2B K120 ubiquitination intermediate mimics through split-intein *trans*-splicing between the E3 enzyme and E2-Ub-conjugated nucleosomes.

To overcome the challenge of ligating large protein segments at low reaction concentrations, we and others have developed templated ligation strategies that bring the reacting partners into physical proximity to increase the effective molarity of the reactants, thereby enabling ligation to occur even at micro- to nanomolar concentrations. Both noncovalent interactions (e.g., peptide nucleic acid interactions,^150,151^ DNA pairing interactions,^152^ electrostatic interactions,^153^ and streptavidin-desthiobiotin binding^154^) and covalent templates (e.g., click chemistry^155^ and split-intein splicing chemistry^156^) have been examined. Nonetheless, to synthesize human proteoforms exceeding 50 kDa in size, a more practical approach is to achieve the individual synthesis and folding of the protein domains of the target protein first, followed by transpeptidative domain–domain ligation under nondenaturing conditions (Figure 6a). Human proteins are usually composed of two or more stable globular domains connected by other nonstructural regions.^157^ The length of a human protein structural domain typically ranges from 50 to 200 amino acids,^158−161^ with most (87.6% of Pfam domains; 95.2% of ECOD domains) of these domains being less than 300 amino acids in length (Table 2).^162^ Transpeptidative protein domain–domain ligation is expected to mitigate the challenge of synthesizing large human proteins that are difficult to fold. In addition, ligation between protein domains in relatively flexible regions offers a solution to address the challenges posed by buried ligation sites.

**Table 2:** Protein Structural Domain Lengths in Human Proteome

structural domain length (aa)	Pfam domain		ECOD domain	
	number	percentage	number	percentage
0–99	2,568	38.7%	786	46.6%
100–199	2,311	34.8%	617	36.6%
200–299	935	14.1%	203	12.0%
300–399	406	6.1%	51	3.0%
400–499	186	2.8%	17	1.0%
500–599	107	1.6%	8	0.5%
600–699	48	0.7%	1	0.1%
700–799	31	0.5%	1	0.1%
≥800	42	0.6%	1	0.1%
total	6,634	100%	1,685	100%

To forge a peptide bond between the two separate protein domains in a chemoselective manner with fast kinetics, one solution is to use enzyme-mediated transpeptidative ligation wherein transpeptidases (e.g., sortase A^163−169^) and proteases (e.g., subtiligase^170^) can be used to induce the formation of a thioester or oxoethyl ester intermediate from one protein segment that can be attacked by an amine donor from another protein segment.^34,171^ Moreover, the split-intein-based protein splicing technique provides a powerful means to convergently ligate synthetic and recombinant protein segments together to make PTM-bearing proteins suitable for mechanistic investigations.^168,172−178^ As a recent example, we exploited a unique Cys-free gp41–1^179^ split intein to synthesize E3/E2∼Ub/NCP as a stable intermediate mimic to elucidate the mechanisms underlying H2B Lys120 monoubiquitination catalyzed by yeast Bre1 or human RNF20/40 E3 ligases (Figure 6b).^172^ The gp41–1 split intein, which undergoes a specific oxoethyl but not thioester intermediate during trans-splicing, was selected for its compatibility with disulfide bond formation between the E2 active center Cys and H2BK120C. The substrate histone H2BK120C, the ubiquitin E2 enzyme Rad6, and ubiquitin were recombinantly expressed and assembled by chemical protein synthesis, followed by folding into E2-Ub conjugated nucleosomes.^82,172^ The E3 ubiquitin ligase Bre1 was subsequently added to facilitate protein splicing between the N-terminal and C-terminal segments of the gp41–1 split intein^180^ at E3 and E2, leading to the close physical proximity of the E3 and E2 enzymes. The protein domain–domain ligation of Bre1 and Rad6 by the split-intein method enabled the structural elucidation of the complete E3/E2∼Ub/nucleosome complex,^172,181,182^ in which the ligation product of 2,907 amino acid residues effectively mimicked the transient ternary intermediate formed during the transfer of Ub from Rad6 to the nucleosome at H2B K120 (Figure 6b).

## Application of Chemical Protein Synthesis to Understand Human Proteoform Biology

A comprehensive understanding of the overall composition and function of the human proteome^183^ has increasingly been recognized as crucial for understanding the mechanisms of disease development, as this would provide support for the development of precision medicine and personalized treatment.^184−187^ The ability to chemically synthesize specific structurally defined human proteoforms enables access to homogeneous samples or customized probes for the study of PTM-associated protein–protein interactions (PPIs), biological function, structural mechanism, drug discovery and development.

### Dissection and Reconstitution of the Interaction Network of Human Proteoforms

**Figure 7:**
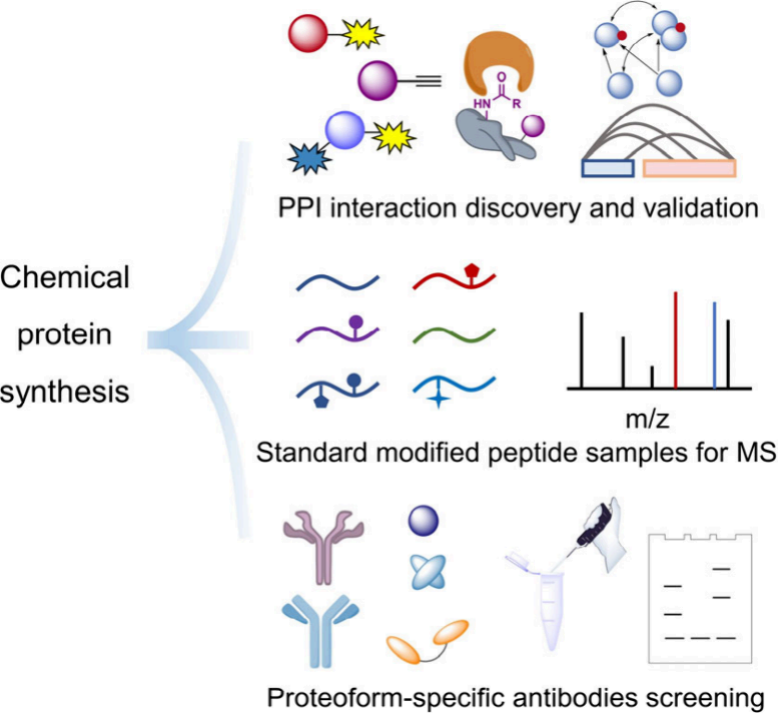
Chemical protein synthesis for dissection and reconstitution of the interaction network of human proteoforms.

The intricate web of human proteoform interactions plays a central role in controlling biological function and the onset and progression of major clinical diseases.^188^ Holistic insights into cellular organization and function necessitate a comprehensive understanding of the interaction networks of different proteoforms in humans. Current PPI identification techniques, such as yeast two-hybrid and expressed protein bait pull-down techniques,^189,190^ can primarily provide information at the protein level, which is insufficient for comprehensively understanding the dynamic interactions and regulatory patterns of human proteoforms, the integral agents responsible for orchestrating vital biological processes. Chemical protein synthesis offers a powerful means of creating modified protein samples with known PTMs and other variations and is essential for the verification and quantification of PPIs. Stable-isotope-labeled, standard-modified peptide samples can be used in mass spectrometry-based proteomics to identify and characterize specific proteoforms with high precision. By comparing the mass spectrometry data with known modifications of the synthesized proteins, researchers can gain valuable insights into the proteoforms present in a sample. Furthermore, the synthesized proteoforms can serve as structurally defined targets for screening and developing proteoform-specific antibodies. As antibodies are crucial tools for selectively detecting and studying specific proteoforms, the ability to generate antibodies against synthetic proteoforms enables a more precise and comprehensive understanding of protein interactions (Figure 7).

Here we use an example to illustrate the utility of chemical protein synthesis in dissecting and reconstituting the interaction network of human proteoforms, wherein we studied ubiquitination, a versatile PTM that regulates a wide range of cellular processes in eukaryotic cells. Through covalent linkage to protein substrates and other ubiquitin molecules, ubiquitin forms complex chains, making specific ubiquitin chain-type antibodies or binders instrumental for understanding ubiquitin biology.^191,192^ Nevertheless, the understanding of K29-linked ubiquitin, despite its prevalence as the most abundant among atypical linkage types accounting for approximately 10% of all linkages in cells,^193^ remains limited due to the dearth of appropriate tools. To bridge this gap, chemical protein synthesis was employed to generate biotinylated K29-linked diUb samples. These samples were then subjected to phage display-based screening, leading to the identification of sAB-K29 - a specific binder exhibiting low nanomolar affinity for K29 chains. To ascertain the binding specificity of sAB-K29, other chemically prepared ubiquitin chain-type samples were used, and the crystal structure of the binder in complex with K29-linked diUb was determined. The sAB-K29 binder was developed for use in immunoprecipitation followed by mass spectrometry, immunoblotting, and immunofluorescence imaging. These techniques enabled the precise detection and validation of K29-linked ubiquitin proteoforms and their interacting partners in cellular and tissue samples. We revealed that the enrichment of K29-linked ubiquitination occurs in response to various proteotoxic cellular stress conditions, including the unfolded protein response. Additionally, K29-linked ubiquitination was found to be enriched in the midbody during the telophase of mitosis. These findings facilitate the dissection of the interaction network of K29-linked ubiquitination in stress responses, protein homeostasis, and cell cycle regulation.^194−196^

### Structural Mechanism Elucidation and Functional Analysis of Human Proteoform Complexes

**Figure 8:**
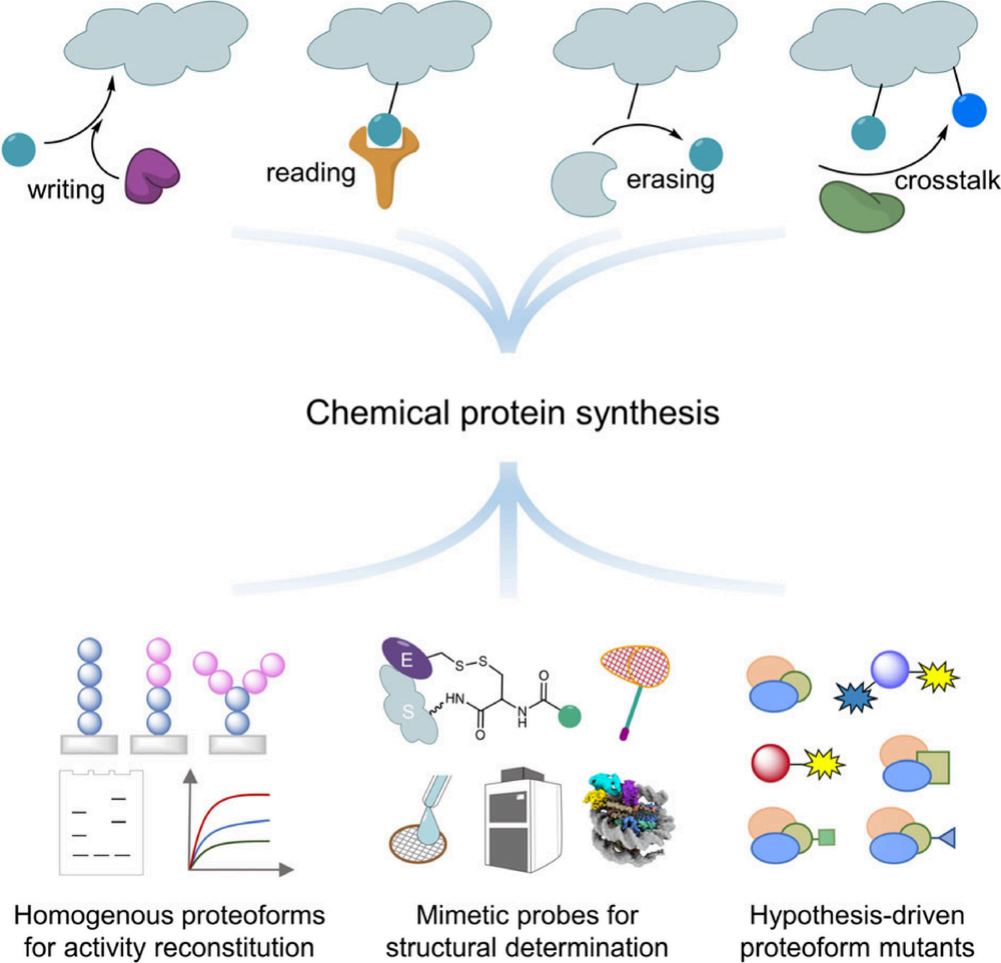
Chemical protein synthesis for structural mechanism elucidation and function analysis of human proteoform complexes.

Deciphering of the intricate code of protein PTMs necessitates quantitative measurements of their biochemical activities and atomic-resolution elucidation of the underlying structural mechanisms. Access to homogeneously modified human proteoforms for mechanistic biochemical elucidation has necessitated the use of chemical protein synthesis.^197−200^ Moreover, the enzymatic processes involved in PTMs are usually transient and dynamic, and it is challenging to obtain stable enzyme–substrate complexes by traditional methods, such as protein complex incubation. Chemical protein synthesis offers a unique solution to enable the development of chemical trapping probes that can stabilize the transient intermediates of enzymatic reactions by constructing stable intermediate analogs that mimic catalytic processes and facilitate structural elucidation.^82,201,202^ Finally, chemical protein synthesis enables the incorporation of nonnatural modifications inaccessible to traditional biological methods, and such tailored modifications can provide a more comprehensive understanding of the structure–activity relationships of proteoforms (Figure 8).

A typical example that illustrates how chemical protein synthesis facilitates mechanistic exploration is the study of the mechanism underlying the activation of the H3K79 methyltransferase Dot1L by H2BK34Ub.^203^ The enzymatic activity of Dot1L, a drug target for mixed-lineage leukemia, is stimulated by nucleosome modifications such as H2BK120Ub,^204−207^ H2BK34Ub,^203,208,209^ and H4K16ac.^210^ In our recent study on the activation of Dot1L activity by H2BK34Ub, histone H2BK34Ub samples, which cannot be obtained through recombinant expression or enzymatic ubiquitination, played a vital role in reconstituting the biochemical activity and in mechanistic investigations. To obtain the H2BK34Ub samples, we employed a three-segment synthesis strategy. First, we recombinantly expressed Ub(1–75)MesNa, and synthesized the H2B(1–57) peptide with an acid-sensitive auxiliary at Lys 34 and the H2B(58–125) peptide through Fmoc-SPPS. Next, the three segments were assembled using peptide hydrazide ligation, followed by desulfurization, affording the desired H2BK34Ub molecule. Chemically synthesized H2BK34Ub was incorporated into nucleosomes and utilized for the biochemical reconstitution of Dot1L activity. Additionally, we determined the structure of the Dot1L-H2BK34Ub nucleosome complex and revealed a unique nucleosome distortion activation mechanism that is independent of enzyme-ubiquitin interactions. To support our findings, we also designed hypothesis-driven mutants, such as H2BK34ac, H2BK34-(aeea)_2_-Ub, and H2BK34sumo, which can only be generated by chemical protein synthesis. These mutants verified the importance of steric hindrance caused by ubiquitin at the H2BK34 site for Dot1L activation.^203^ Thus, the H2BK34Ub-Dot1L study, as well as many other studies showed the importance of chemical protein synthesis in understanding the structural mechanisms underlying the effects of proteoform complexes.^211,212^

### Development and Evaluation of Drugs Targeting Human Proteoforms

**Figure 9:**
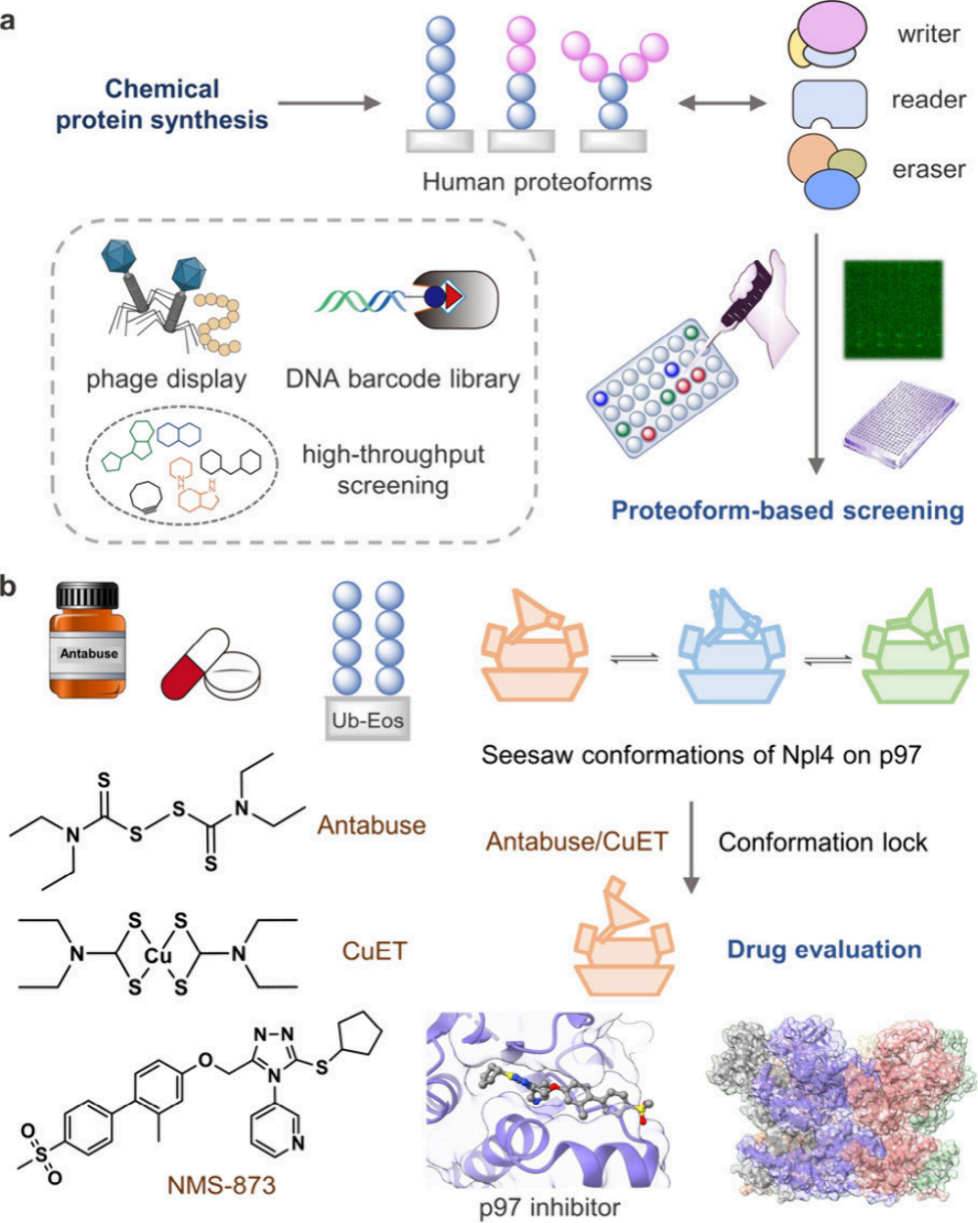
Development and evaluation of drugs targeting human proteoforms. (a) Chemical protein synthesis enables the generation of precisely modified human proteoforms, which can be utilized for proteoform-based screening of bioactive molecules. (b) Exemplary chemical regulators, such as Antabuse, CuET, and NMS-873 that targets p97, with their respective working mechanisms illustrated.

An important aspect of the development of drugs that target proteoform interactions is the establishment of human proteoform-based activity screening assays. These assays are essential for accurately screening chemical regulators or drugs that target proteoform-specific enzymes involved in writing, erasing, and reading. Access to proteoform substrates is imperative for precise evaluation and chemical protein synthesis offers the ability to provide homogeneous modified proteoform samples for these assays, where a library of compounds can be screened to identify potential drug candidates with the desired biological activities^213,214^ (Figure 9a). For instance, nucleosomal deubiquitinases (such as USP16,^215^ PR-DUB,^216,217^ and the SAGA DUB module^218^) are increasingly being recognized as highly promising drug targets. When screening small molecules to suppress or activate deubiquitination activity, conventional model substrates such as fluorescent ubiquitin 7-amido-4-methylcoumarin (Ub-AMC) or ubiquitin-Rhodamine 110 (Ub-Rho) are typically employed.^218^ Using site-specifically ubiquitinated nucleosomes (e.g., H2AK119Ub and H2BK120Ub) as screening substrates would enhance the possibility of identifying regulators that are specifically targeted to substrates.

Another utility of chemical protein synthesis is the understanding of the molecular mechanism by which drug molecules regulate human proteoforms, thereby enhancing structure–activity analysis and rational design of more effective drugs. In our recent mechanistic studies of the antialcohol abuse drug disulfiram (an FDA-approved drug with the trade name Antabuse)^219^ and the disulfiram metabolic derivative CuET which has been found to bind to zinc finger 1 of the human p97 cofactor Npl4 and exhibit broad antitumor activity.^220,221^ We chemically synthesized the fluorescent K48-polyubiquitinated substrate Ub-Eos using the gp78^RING^-Ube2g2 E3-E2 enzyme pair.^219^ The products were used for unfolding activity experiments, which revealed that p97 activity was inhibited by the divalent copper ions released from CuET rather than by CuET itself. The ubiquitinated substrates were also applied for structural studies of p97 in complex with its cofactors Ufd1/Npl4 and Ub-Eos in the presence or absence of CuET, revealing seesaw conformations of Npl4 in the p97 complex, with the seesaw conformation locked in a specific state in the presence of the disulfiram derivative CuET (Figure 9b).^219^ Furthermore, synthetic Ub-Eos samples were also used for biochemical activity testing and structural determination of the allosteric inhibitor of p97, NMS-873, which has broad antiviral activity. The results showed that NMS-873 binds to a hidden pocket surrounding the ISS motif of the p97 D2 domain, disrupting the asymmetric unfolded state of p97 (Figure 9b).^222^ These studies illustrate the usefulness of using chemically synthesized proteins to study how bioactive molecules interact with human proteoform complexes, and knowledge gained from such studies may aid in the development of improved drug molecules.

## Outlook: Opportunity and Challenges

**Figure 10:**
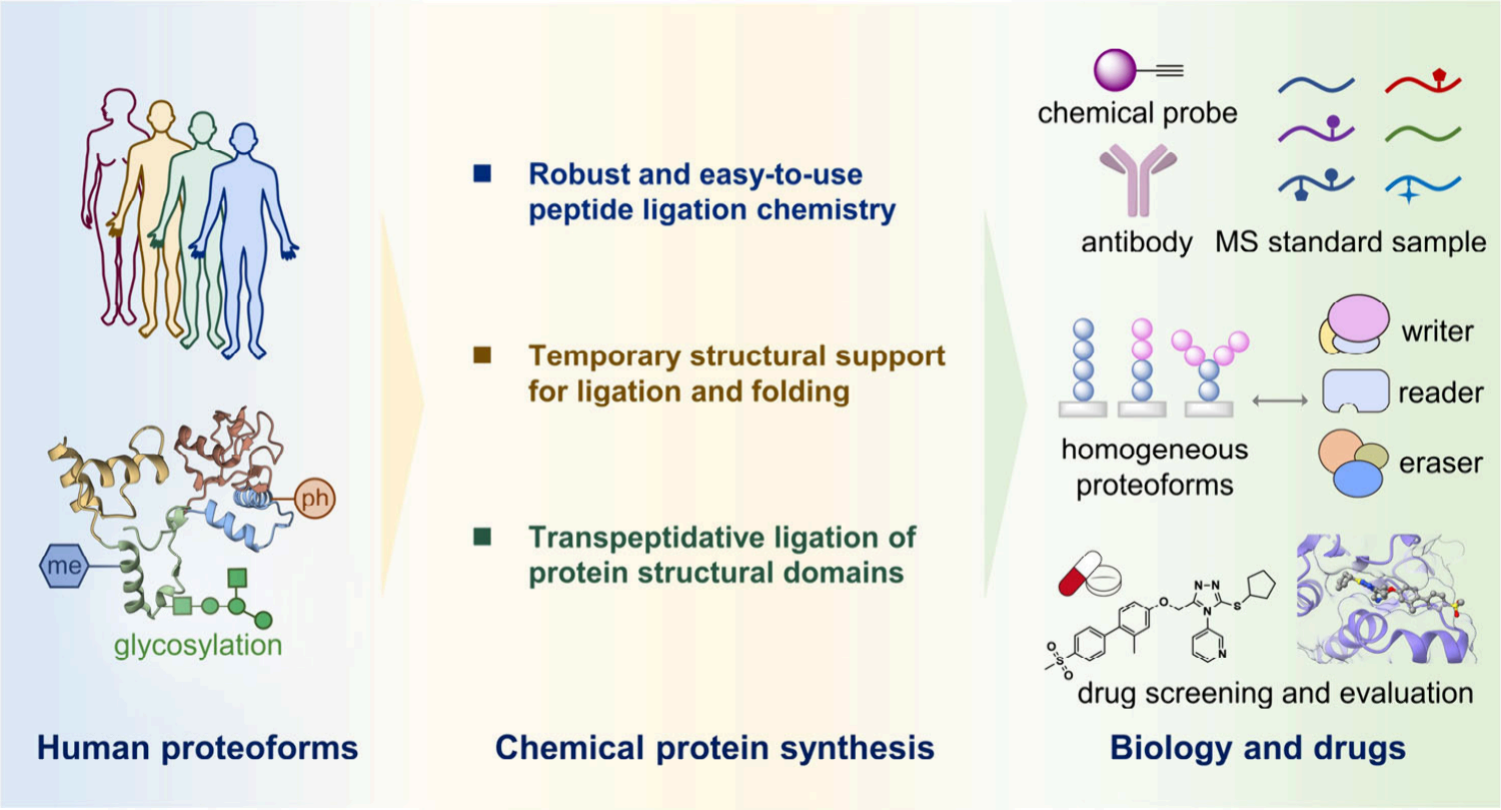
Summary and outlook for the chemical protein synthesis of human proteoforms.

In the past years we have been trying to develop a comprehensive, robust, and cost-effective methodology for the chemical synthesis of all human proteoforms, which entails three synthetic strategies: 1) easy-to-use peptide ligation chemistries that allow more efficient *de novo* synthesis of any protein structural domains; 2) robust strategies for providing temporary structural support for the ligation and folding of more challenging protein targets; and 3) efficient transpeptidative protein domain−domain ligation methods for the synthesis of multidomain proteins. Using these methods, we have synthesized a variety of structurally defined and homogeneous human proteoforms and used them for the dissection and reconstitution of intricate interaction networks of human proteoforms, the elucidation of the structural mechanisms and functional roles of human proteoform complexes, and the development and evaluation of drugs targeting human proteoforms. These studies have indicated that chemical protein synthesis can play an important role in research on human health and disease and facilitate the development of precision diagnostics and therapeutics (Figure 10).

### Limitation of the Current Chemical Protein Synthesis Techniques and Potential Solutions

Traditional biological approaches like recombinant expression or cellular extraction often struggle to provide proteoform samples with the structurally precise and defined PTMs necessary for research. The inherent heterogeneity or limitations of these methods make it challenging to obtain the highly pure, homogeneous proteoforms required for in-depth studies. In contrast, chemical protein synthesis offers a potent *de novo* construction approach. By incorporating desired PTM-carrying building blocks, and then performing precise peptide or protein domain ligation and refolding, this methodology provides access to well-defined proteoform samples that are otherwise difficult to obtain through traditional biological techniques. This approach furnishes a valuable tool for advancing biomedicine research.

However, chemical protein synthesis does face some inherent limitations. The most prominent challenge is the scalability and efficiency of synthesis, particularly for large, complex protein domains. The stepwise nature of chemical ligation and the potential for side reactions or incomplete modifications can lead to lower yields and purity as the target protein size increases. Additionally, the proper refolding of larger, multidomain proteins remains a technical hurdle. To address these limitations, future research should explore strategies such as automated synthesis platforms, computational design of optimal ligation schemes, and the development of new building blocks and catalysts to streamline PTM incorporation. Advancements in analytical techniques for rapid characterization of synthetic proteoforms will also be crucial to validate the accuracy and fidelity of the chemical synthesis approach.

There are still many challenges in the pursuit of chemical synthesis of the entire human proteome. The diverse PTMs on human proteoforms place greater demands on the development of site-selective and accurate PTM installation methods. For large molecular weight proteoforms (over 100 kDa), scalability and cost would be considerable aspects. A collaborative approach involving chemically synthesized protein domains bearing PTMs combined with expressed protein domains without PTMs could be a strategy to address this. The quality control and verification of the synthesized proteoforms will also require attention, utilizing interdisciplinary characterization techniques. As a risk-mitigating measure, a nonmodified sample should be synthesized first and compared with the recombinant counterpart; this practice could validate the synthetic routine. The future research directions are anticipated to focus on the development of highly efficient and scar-less transpeptidative ligation of protein domains under nondenaturing conditions. This approach is feasible, as most protein structural domains are less than 300 amino acids in length and can be efficiently synthesized using current ligation and refolding strategies.

### Artificial Intelligence (AI) and Chemical Protein Synthesis

Recently, advancements in artificial intelligence have been revolutionizing in the field of chemistry and biology, such as the organic synthesis,^223−225^ rational protein design^226−229^ and protein structure prediction.^230−234^ The potential of AI to facilitate the development of chemical protein synthesis remains to be explored. To harness the power of AI in chemical protein synthesis, comprehensive knowledge on peptide and protein synthesis can be incorporated into machine learning models. This includes information about peptide synthesis, peptide ligation, orthogonal protection strategy, desulphurization strategy and enzymatic strategy. By training the AI with this wealth of knowledge, an exceptional protein chemist would be created. Furthermore, AI can leverage physical and chemical statistics, such as solubility, polarity, and structure, obtained from databases like UniProt, the Protein Data Bank (PDB), and the AlphaFold predicted protein database. By integrating this valuable information, AI can aid in designing the most rational route for synthesizing a specific protein. Factors considered in this process include peptide segmentation, peptide ligation scheme, and protein retrosynthesis analysis. The aim is to provide an optimized protein synthesis analysis scheme that offers the shortest steps, shortest time, and highest efficiency.

Hardware development would also be crucial for efficient experimentation in chemical protein synthesis. Currently, there have been advancements in microware-assisted peptide synthesis^235−237^ and flow chemistry-based peptide synthesis.^238−240^ It is anticipated that future developments will lead to the creation of robots capable of automating peptide synthesis, peptide ligation, and analyzing reactions using techniques like high-performance liquid chromatography (HPLC) and mass spectrometry (MS). These robots would also be able to automatically conduct product purification and protein folding. The ultimate goal is to create an integrated protein synthesizer that maximizes productivity.

### Perspective of the Chemical Synthesis of Human Proteoform and Its Application in Biomedicine

Efficiency is a perpetual concern during the chemical protein synthesis of human proteoforms. Although peptide segments can now be readily prepared through automated synthesizers, peptide ligation instruments for automated chemical protein synthesis are still lacking. Additionally, although temporary structural support strategies are now available^128,132^ for protein targets such as transmembrane proteins, modification groups that can be more easily installed and removed await further development. Moreover, whether methods based on oligosaccharides could provide new opportunities for improving both ligation and folding during chemical protein synthesis remains to be examined. Finally, for the chemical synthesis of proteins greater than 50 kDa in size, it is critical to develop more efficient methods of transpeptidative protein domain−domain ligation. Strategies such as conditional protein splicing techniques,^241−243^ orthogonal split-intein pairing,^244^ and tandem protein trans-splicing^245^ hold promise for synthesizing larger, more complex proteins with multiple modifications. It is expected that through the continued development of robust and cost-effective methodologies for the chemical synthesis of human proteoforms, we will be able to study more challenging targets, research on which remains infeasible with the current technology. One such example is the 2442-amino-acid CBP acetyltransferase, an important epigenetic regulator,^246^ that carries a variety of PTMs (methylation, acetylation, phosphorylation, ubiquitination and sumoylation) across its sequence, and still poses a formidable challenge for chemical protein synthesis, which is needed to examine the roles of these PTMs in regulating CBP’s acetyl transfer activity toward histone and nonhistone substrates.

Regarding the applications of accurate chemical synthesis of human proteoforms in contemporary biomedical research, three areas are of special interest. First, for epigenetic events occurring in the cell nucleus, chemical protein synthesis can allow the generation of precisely modified histones or nucleosomes. These proteins can be used to study the interactions of histone modifications with effector proteins such as pioneering transcription factors and chromatin remodelers, as well as biochemical reconstitution and structural elucidation of various “histone code” events (writing, reading, erasing, and functional histone crosstalk).^247^ Moreover, synthetic nucleosomes bearing tailored modifications may allow the screening and evaluation of bioactive molecules targeting epigenetic proteins such as poly(ADP-ribose) polymerase (PARP) in refractory breast and ovarian cancers.^248^ Second, for protein homeostasis events in the cytoplasm, chemical protein synthesis allows the generation of homogeneous ubiquitinated proteins for the development of proteoform-specific antibodies, the production of standard modified peptides to facilitate quantitative mass spectrometry analysis, and the customization of structurally defined protein probes tailored for biochemical and structural studies of the ubiquitin-proteasome system (UPS), which encompasses ubiquitin E3 ligases, deubiquitinases, p97 unfoldase and proteasomes. Such studies would provide valuable opportunities for understanding protein dynamics, folding pathways, and the impact of PTMs (particularly the complex ubiquitination modifications) on protein degradation, as well as for screening and evaluating small-molecule protein degraders for drug development (e.g., molecular glues or PROTACs). Finally, for the signal transmission events in cell membranes, cell membrane proteins (GPCRs, ion channels, etc.) are the main executors of intra- and extracellular substance exchange and signal transmission, and dysfunction in these proteins is directly related to the development of human diseases, with over 50% of currently marketed drugs targeting on membrane proteins.^249^ Chemical protein synthesis can contribute by enabling the preparation of these membrane proteins bearing PTMs, such as phosphorylated and ubiquitinated GPCRs, which are not obtainable through recombinant expression. Such synthetic proteins can facilitate studies on the regulation of GPCR structure and function by PTMs and on how drug molecules interact with these modified GPCRs and their G-protein and β-arrestin complexes. Increased research in the above areas is expected to provide an increasing number of examples showing that chemical protein synthesis is integral to unraveling the complexities of human proteoform biology and the development of precision diagnostics and therapeutics.

## Supporting Information Available

Transparent Peer Review report available (PDF)
