# Supplementary material for: Chemical Synthesis of Human Proteoforms and Application in Biomedicine
Source: ACS Cent Sci. 2024 Jul 22;10(8):1442–59. doi: 10.1021/acscentsci.4c00642 (PMC11363345; doi:10.1021/acscentsci.4c00642)
Supplement: Supplementary file 1 — oc4c00642_si_001.pdf [file oc4c00642_si_001.pdf]

oc-2024-006423.R1

Name: Peer Review Information for "Chemical Synthesis of Human Proteoforms and Application in Biomedicine"

First Round of Reviewer Comments

Reviewer: 1

Comments to the Author

The manuscript from Liu et al is an Outlook that covers some topics of interest in the general area of chemical protein synthesis. The Outlook covers three main topics, namely the development of ligation strategies that would allow for facile assembly of large complex proteins without unwanted side products, the strategy of using supports to facilitate the folding of more intricate proteins, and the use of catalysts for transpeptidation to allow for assembly. While these are all interesting topics, the Outlook as written will likely have a limited audience appeal. The major limitation of the current document is that it focuses largely on work from the investigators own lab rather than providing a broader context. In addition, the title and abstract as misleading as topics such as the synthesis of post-translationally modified proteins is given limited attention.

There would be an interest in an Outlook that covered the topic of advances in chemical protein synthesis, that covers topics such as post-translational modifications, strategies for introducing novel functionalities, advances in semi-synthesis, as well as highlighting efforts from labs other than those of the writers of the current document. If the authors are able to provide a broader contextual overview of these topics, enthusiasm for such an Outlook would be significantly enhanced.

Reviewer: 2

Comments to the Author

This manuscript reviewed the chemical synthesis of human proteins and the applications in biomedicine. This topic is of interest in protein synthesis and drug discovery. This manuscript was clearly written, and provides many examples from recent studies in this field. I suggest the acceptance of this manuscript after the following issues are addressed.

1. Throughout the text, the authors mentioned that post-translational modifications (PTMs) of proteins are important steps in the synthesis of proteoforms. However, in the subsequent sections of the classification, the authors did not specifically address this process and its methods. Considering the uniqueness of protein site-specific modifications through chemical synthesis, such synthetic methods and their biomedical applications should be specifically introduced.

2. In addition to conventional proteins, proteomes also contain special classes of proteins such as nucleic acid-binding proteins and glycoproteins. It is important for the authors to appropriately mention these protein classes and discuss the current research status of their chemical synthesis.

3. In recent years, significant advancements in protein design have been revolutionizing nearly every field of biomedicine. Protein synthesis, as an important approach to materialize protein design, holds great potential for generating exciting interactions and promising applications. The authors should consider incorporating these aspects in the manuscript, either within the main body or in the outlook section, to attract a wider range of interest.

4. The authors briefly mentioned the limitations of current protein chemical synthesis techniques. However, it is important to appropriately address the physical limitations of current technologies, as well as the underlying reasons for these limitations and potential solutions.

Reviewer: 3

Comments to the Author

This paper effectively addresses a critical challenge in human health research: understanding the function of complex human proteoforms. The authors outline the key chemical advancements needed for efficient and precise synthesis of these molecules, including novel peptide ligation strategies for PTM incorporation, temporary structural support for folding, and efficient domain ligation methods. They further explore the applications of chemically synthesized proteoforms in dissecting protein-protein interaction networks, elucidating structures and functions of proteoform complexes, and developing drugs targeting specific proteoforms. By highlighting the need for new chemistries, the paper lays out a roadmap for future advancements in this transformative field.

Here are some additional points the authors might consider including in this paper:

1. Briefly mentioning the vast number of human proteoforms due to PTM combinations. This emphasizes the need for efficient and scalable chemical synthesis methods.

2. Briefly discussing the limitations of current methods for studying proteoforms (e.g., recombinant expression) and how chemical synthesis offers advantages.

3. Addressing some of the main challenges in chemically synthesized proteoforms, such as the complexity of PTM incorporation, scalability and cost considerations, verification and quality control methods, and future research directions in this field.

Therefore, I recommend a minor revision for this paper to be considered for acceptance.

Author's Response to Peer Review Comments:

Lei Liu, Professor
Department of Chemistry

Tsinghua University
Beijing 100084, P. R. China
Tel: 86-10-62780027
Fax: 86-10-62771149
Email: lliu@mail.tsinghua.edu.cn

Jun 27, 2024

Manuscript Title: Chemical Synthesis of Human Proteoforms and Application in Biomedicine (Manuscript ID: oc-2024-006423)

Corresponding author: Lei Liu, Prof. Dr.

Dear Editor

Thank you very much for your kind consideration of our manuscript. We have carefully made changes according to the suggestions of the reviewers and formatting needs in ACS Central Science (as marked in yellow in the revised manuscript):

Formatting Needs:

MS HEADER: The manuscript should be formatted with a cover sheet listing authors, author affiliations, corresponding author email, manuscript title, and the number of pages, figures, and tables. The Author affiliations must match the SI.

Response: We have provided the author affiliations, corresponding author email in the cover page, we hope to meet the journal's formatting requirements.

ABSTRACT WORD COUNT: Please make sure the word count does not exceed 200 words.

Response: The Abstract has been abbreviated within 200 words.

PULL QUOTE (OUTLOOKS + IN FOCUS): Please select 3 - 4 quotes from your submission that you would like highlighted in your paper. The quotes should be one sentence-long, unique to the submission and not from previously cited work. Please list your quotes at the end of the manuscript file.

Response: Four one-sentence quotes have been selected and listed at the end of the manuscript.

SYNOPSIS MISSING: The synopsis should be no more than 200 characters (including spaces) and should reasonably correlate with the TOC graphic. The synopsis is intended to explain the importance of the article to a broader readership across the sciences. Please place your synopsis in the manuscript file after the TOC graphic, and label it as "Synopsis."

Response: We have provided a Synopsis in the manuscript after the TOC graphic with a length of no more than 200 characters including spaces.

TOC: Please upload as "Graphic for Manuscript," and move to the last page of the manuscript with a label "TOC Graphic."

Response: As required, we have provided the TOC in the last page of the manuscript and uploaded the TOC file as "Graphic for Manuscript".

Reviewer: 1

Recommendation: Reconsider after major revisions noted.

Response: We sincerely appreciate the reviewer for taking the time to review our manuscript. We have carefully considered the reviewer's comments and have made significant revisions to address them. Below, we provide detailed responses to the reviewer's comments.

Comments:

The manuscript from Liu et al is an Outlook that covers some topics of interest in the general area of chemical protein synthesis. The Outlook covers three main topics, namely the development of ligation strategies that would allow for facile assembly of large complex proteins without unwanted side products, the strategy of using supports to facilitate the folding of more intricate proteins, and the use of catalysts for transpeptidation to allow for assembly.

Response: We thank the reviewer for the comments.

While these are all interesting topics, the Outlook as written will likely have a limited audience appeal. The major limitation of the current document is that it focuses largely on work from the investigators own lab rather than providing a broader context. In addition, the title and abstract as misleading as topics such as the synthesis of post-translationally modified proteins is given limited attention. There would be an interest in an Outlook that covered the topic of advances in chemical protein synthesis, that covers topics such as post-translational modifications, strategies for introducing novel functionalities, advances in semi-synthesis, as well as highlighting efforts from labs other than those of the writers of the current document. If the authors are able to provide a broader contextual overview of these topics, enthusiasm for such an Outlook would be significantly enhanced.

Response: We thank the reviewer for this valuable suggestion and have made several enhancements. Specifically, we have added a new paragraph to the Introduction section describing strategies for generating post-translationally modified proteins, including genetic code expansion techniques, bio-orthogonal functionalization methods, chemoenzymatic strategies, and *de novo* chemical protein synthesis, as illustrated in the newly added **Figure 2**. We have also provided additional citations to show respect for the work of other laboratories.

*"In this context, chemical strategies have been developed to produce proteoforms with site-specific modifications First, genetic code expansion techniques enable the ribosomal integration of unnatural amino acids into proteins within living cells. The unnatural amino acids can carry modifications such as acylation and phosphorylation[11-13], while more complex PTMs (e.g. glycosylation, ubiquitination) remain difficult to incorporate due to challenge in the engineering of ribosomal aminoacyl-tRNA synthetases (**Figure 2a**). Second, bio-orthogonal functionalization techniques enable the preparation of site-selectively modified proteins at specific residues such as Cys[14-17], [18, 19]. Lys[20-22], Met[23-28], and Trp[29-32] of expressed protein (**Figure 2b**). PTM mimics are usually produced through this technology, which however, may occasionally result in unforeseen biological consequences or ambiguity in interpreting experimental findings[33]. Third, chemoenzymatic methods[34-36] can be employed for obtaining proteoform samples where PTM enzymes (e.g. E3 ligase[37, 38]) or other engineered enzymes (e.g., Sortase A[39-41], Butelase-1[42]) enable protein modification with site-selectivity (**Figure 2c**). This approach falls short for modifications lacking known enzymes or when the existing enzymes do not have sufficient substrate specificity. Finally, de novo chemical protein synthesis, a bottom-up approach for the construction of proteins, principally allows non-natural amino acids to be incorporated into a protein at any positions, and in any numbers and combinations[43-45]. This approach could be more difficult to carry out, but it provides an avenue to complement the other methods for generating proteoforms with precise structures at atomic resolution[43, 46, 47] (**Figure 2d, 3**)."*

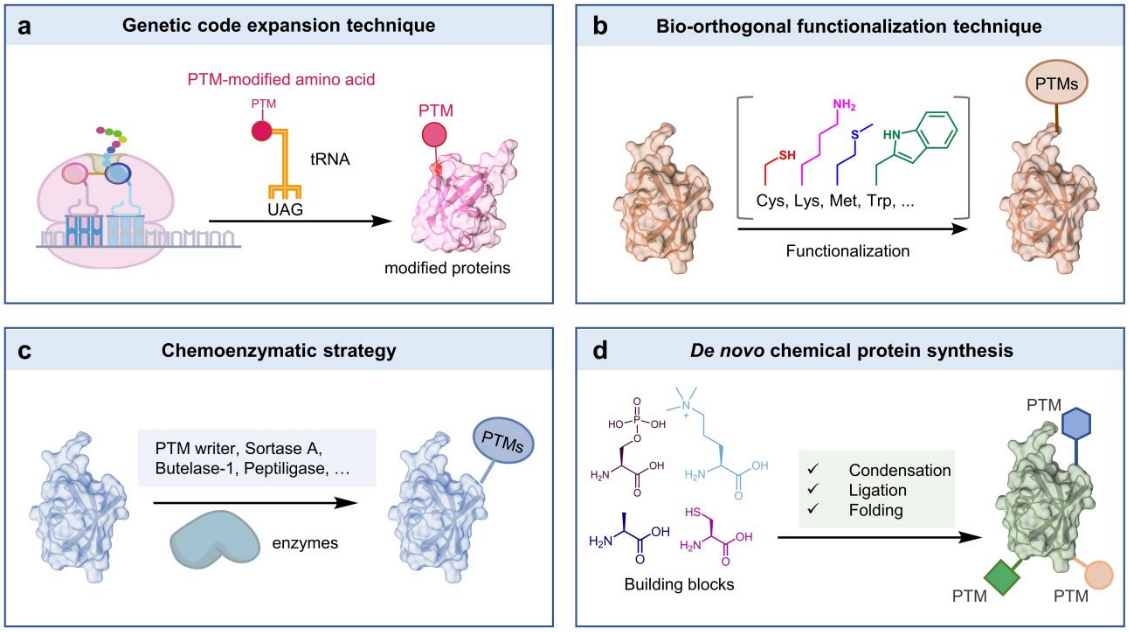

Figure 2. Synthesis of post-translationally modified proteins or proteoforms. *Current four strategies can be used to prepare PTM proteins: genetic code expansion technique, bio-orthogonal functionalization technique, chemoenzymatic strategy, and de novo chemical protein synthesis.*

Furthermore, to broaden the appeal of our manuscript, as suggested by reviewer 2, we have expanded the discussion on the potential applications of artificial intelligence (AI) and protein design in chemical protein synthesis. This includes both software and hardware aspects, aimed at optimizing synthetic schemes for a given protein to achieve minimal steps, time efficiency, and maximum productivity. Additionally, we discuss the concept of an integrated protein synthesizer designed to enhance overall efficiency.

*"**Artificial intelligence (AI) and chemical protein synthesis.***

Recently, advancements in artificial intelligence have been revolutionizing in the field of chemistry and biology, such as the organic synthesis[223-225], rational protein design[226-229] and protein structure prediction[230-234]. The potential of AI to facilitate the development of chemical protein synthesis remains to be explored. To harness the power of AI in chemical protein synthesis, comprehensive knowledge on peptide and protein synthesis can be incorporated into machine learning models. This includes information about peptide synthesis, peptide ligation, orthogonal protection strategy, desulphurization strategy and enzymatic strategy. By training the AI with this wealth of knowledge, an exceptional protein chemist would be created. Furthermore, AI can leverage physical and chemical statistics, such as solubility, polarity, and structure, obtained from databases like UniProt, the Protein Data Bank (PDB), and the AlphaFold predicted protein database. By integrating this valuable information, AI can aid in designing the most rational route for synthesizing a specific protein. Factors considered in this process include peptide segmentation, peptide ligation scheme, and protein retrosynthesis analysis. The aim is to provide an optimized protein synthesis analysis scheme that offers the shortest steps, shortest time, and highest efficiency.

Hardware development would also be crucial for efficient experimentation in chemical protein synthesis. Currently, there have been advancements in microware-assisted peptide synthesis[235-237] and flow chemistry-based peptide synthesis[238-240]. It is anticipated that future developments will lead to the creation of robots capable of automating peptide synthesis, peptide ligation, and analyzing reactions using techniques like high-performance liquid chromatography (HPLC) and mass spectrometry (MS). These robots would also be able to automatically

conduct product purification and protein folding. The ultimate goal is to create an integrated protein synthesizer that maximizes productivity."

Our manuscript aims to provide a forward-looking perspective rather than a traditional literature review, which limits the length of the manuscript. We therefore only included essential information to provide the reader with a conceptual understanding of these strategies. Alongside revisions based on feedback from other reviewers, we hope that these amendments meet the reviewer's expectations and enrich the manuscript's value to a broader audience interested in the future directions of chemical protein synthesis.

Additional Questions:
Quality of experimental data, technical rigor: High
Significance to chemistry researchers in this and related fields: High
Broad interest to other researchers: High
Novelty: High
Is this research study suitable for media coverage or a First Reactions (a News & Views piece in the journal)?: No

We thank the reviewer again for the comments and valuable suggestions that are very helpful for improving the quality of our manuscript.

Reviewer: 2

Recommendation: Publish in ACS Central Science after minor revisions noted.
Response: We thank the reviewer for supporting the publication of our manuscript.

Comments:
This manuscript reviewed the chemical synthesis of human proteins and the applications in biomedicine. This topic is of interest in protein synthesis and drug discovery. This manuscript was clearly written, and provides many examples from recent studies in this field. I suggest the acceptance of this manuscript after the following issues are addressed.
Response: We thank the reviewer for the positive comments.

1. Throughout the text, the authors mentioned that post-translational modifications (PTMs) of proteins are important steps in the synthesis of proteoforms. However, in the subsequent sections of the classification, the authors did not specifically address this process and its methods. Considering the uniqueness of protein site-specific modifications through chemical synthesis, such synthetic methods and their biomedical applications should be specifically introduced.
Response: We acknowledge the feedback regarding the initial omission of specific methods for synthesizing PTM-modified proteins. In response, we have elaborated on this in the Introduction section and provided a visual representation of these methods in the newly-provided **Figure 2**, which includes genetic code expansion techniques, bio-orthogonal functionalization methods, chemoenzymatic strategies, and de novo chemical protein synthesis.

*"In this context, chemical strategies have been developed to produce proteoforms with site-specific modifications First, genetic code expansion techniques enable the ribosomal integration of unnatural amino acids into proteins within living cells. The unnatural amino acids can carry modifications such as acylation and phosphorylation[11-13], while more complex PTMs (e.g. glycosylation, ubiquitination) remain difficult to incorporate due to challenge in the engineering of ribosomal aminoacyl-tRNA synthetases (**Figure 2a**). Second, bio-orthogonal functionalization techniques enable the preparation of site-selectively modified proteins at specific residues such as Cys[14-17, 18, 19]. Lys[20-22], Met[23-28], and Trp[29-32] of expressed protein (**Figure 2b**). PTM mimics are usually produced through this technology, which however, may occasionally result in unforeseen biological consequences or ambiguity in interpreting experimental findings[33]. Third, chemoenzymatic methods[34-36] can be employed for obtaining proteoform samples where PTM enzymes (e.g. E3 ligase[37, 38]) or other engineered enzymes (e.g., Sortase A[39-41], Butelase-1[42]) enable protein modification with site-selectivity (**Figure 2c**). This approach falls short for modifications lacking known enzymes or when the existing enzymes do not have sufficient substrate specificity. Finally, de novo chemical protein synthesis, a bottom-up approach for the construction of proteins, principally allows non-natural amino acids to be incorporated into a protein at any positions, and in any numbers and combinations[43-45]. This approach could be more difficult to carry out, but it provides an avenue to complement the other methods for generating proteoforms with precise structures at atomic resolution[43, 46, 47] (**Figure 2d, 3**)."*

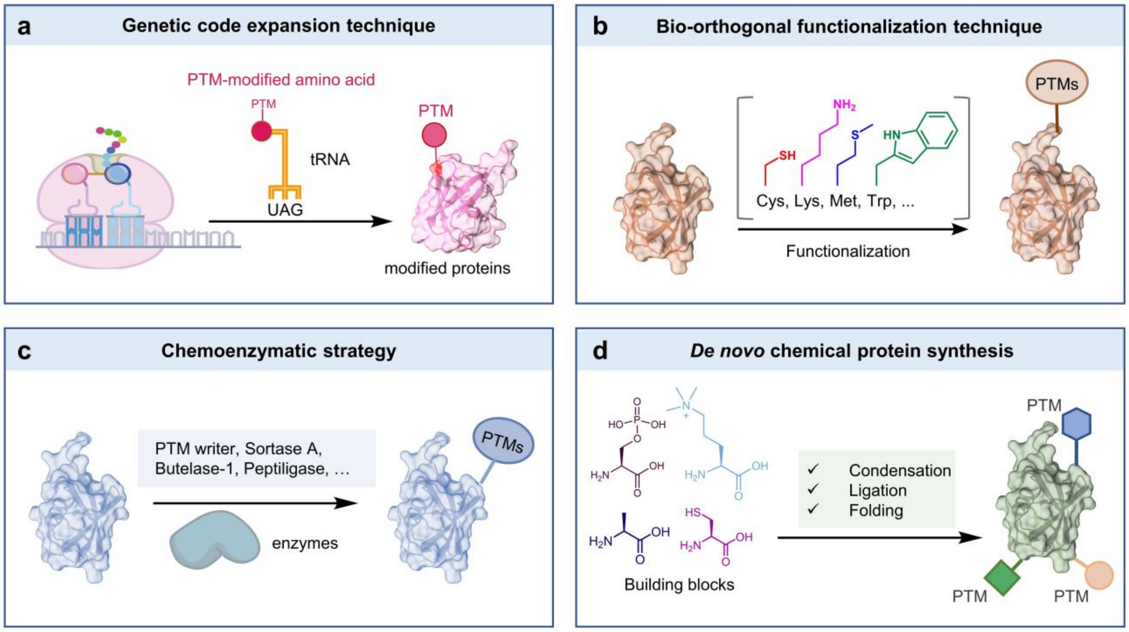

Figure 2. Synthesis of post-translationally modified proteins or proteoforms. *Current four strategies can be used to prepare PTM proteins: genetic code expansion technique, bio-orthogonal functionalization technique, chemoenzymatic strategy, and de novo chemical protein synthesis.*

2. In addition to conventional proteins, proteomes also contain special classes of proteins such as nucleic acid-binding proteins and glycoproteins. It is important for the authors to appropriately mention these protein classes and discuss the current research status of their chemical synthesis.

Response: We thank the reviewer for this valuable suggestion and have incorporated the chemical synthesis of special classes of proteins such as histones, nucleic acid-binding protein, lipidated proteins and glycoproteins in our revised manuscript.

"These strategies have advanced the synthesis of small to medium-sized human proteoforms, such as the core histone protein (H2A, H2B, H3 and H4 with residues ranging from 100~130) carrying complex types and combinations of PTMs[111-115], the nucleic acid-binding proteins like 160-residue transcription factor protein MAX/MYC with patterns of phosphorylation and acetylation[116, 117], lipidated proteins like palmitoylated 178-residue caveolin-1[118, 119], and glycoproteins like 312-residue ribonuclease B[120-124]."

3. In recent years, significant advancements in protein design have been revolutionizing nearly every field of biomedicine. Protein synthesis, as an important approach to materialize protein design, holds great potential for generating exciting interactions and promising applications. The authors should consider incorporating these aspects in the manuscript, either within the main body or in the outlook section, to attract a wider range of interest.

Response: We appreciate the reviewer's valuable suggestion, which would undoubtedly improve the overall quality and scope of our manuscript. In our revised manuscript, we have included this in the Outlook section, subtitled "**Artificial intelligence (AI) and chemical protein synthesis**".

*"**Artificial intelligence (AI) and chemical protein synthesis.***

Recently, advancements in artificial intelligence have been revolutionizing in the field of chemistry and biology, such as the organic synthesis[223-225], rational protein design[226-229] and protein structure prediction[230-234]. The potential of AI to facilitate the development of chemical protein synthesis remains to be explored. To harness the power of AI in chemical protein synthesis, comprehensive knowledge on peptide and protein synthesis can be incorporated into machine learning models. This includes information about peptide synthesis, peptide ligation, orthogonal protection strategy, desulphurization strategy and enzymatic strategy. By training the AI with this wealth of knowledge, an exceptional protein chemist would be created. Furthermore, AI can leverage physical and chemical statistics, such as solubility, polarity, and structure, obtained from databases like UniProt, the Protein Data Bank (PDB), and the AlphaFold predicted protein database. By integrating this valuable information, AI can aid in designing the most rational route for synthesizing a specific protein. Factors considered in this process include peptide segmentation, peptide ligation scheme, and protein retrosynthesis analysis. The aim is to provide an optimized protein synthesis analysis scheme that offers the shortest steps, shortest time, and highest efficiency.

Hardware development would also be crucial for efficient experimentation in chemical protein synthesis. Currently, there have been advancements in microware-assisted peptide synthesis[235-237] and flow chemistry-based peptide synthesis[238-240]. It is anticipated that future developments will lead to the creation of robots capable of automating peptide synthesis, peptide ligation, and analyzing reactions using techniques like high-performance liquid chromatography (HPLC) and mass spectrometry (MS). These robots would also be able to automatically conduct product purification and protein folding. The ultimate goal is to create an integrated protein synthesizer that maximizes productivity."

4. The authors briefly mentioned the limitations of current protein chemical synthesis techniques. However, it is important to appropriately address the physical limitations of current technologies, as well as the underlying reasons for these limitations and potential solutions.

Response: We agree with the reviewer and have taken the opportunity to include this consideration in the Outlook section of our revised manuscript, entitled "**Limitation of the current chemical protein synthesis techniques and potential solutions**", where we have provided a comprehensive discussion of the physical limitations of current technologies. We have also highlighted the underlying reasons for these limitations and presented potential solutions to overcome them.

*"**Limitation of the current chemical protein synthesis techniques and potential solutions.***

Traditional biological approaches like recombinant expression or cellular extraction often struggle to provide proteoform samples with the structurally precise and defined PTMs necessary for research. The inherent heterogeneity or limitations of these methods make it challenging to obtain the highly pure, homogeneous proteoforms required for in-depth studies. In contrast, chemical protein synthesis offers a potent de novo construction approach. By incorporating desired PTM-carrying building blocks, and then performing precise peptide or protein domain ligation and refolding, this methodology provides access to well-defined proteoform samples that are otherwise difficult to obtain through traditional biological techniques. This approach furnishes a valuable tool for advancing biomedicine research.

However, chemical protein synthesis does face some inherent limitations. The most prominent challenge is the scalability and efficiency of synthesis, particularly for large, complex protein domains. The step-wise nature of chemical ligation and the potential for side reactions or incomplete modifications can lead to lower yields and purity as the target protein size increases. Additionally, the proper refolding of larger, multi-domain proteins remains a technical hurdle. To address these limitations, future research should explore strategies such as automated synthesis platforms, computational design of optimal ligation schemes, and the development of new building blocks and catalysts to streamline PTM incorporation. Advancements in analytical techniques for rapid characterization of synthetic proteoforms will also be crucial to validate the accuracy and fidelity of the chemical synthesis approach.

There are still many challenges in the pursuit of chemical synthesis of the entire human proteome. The diverse PTMs on human proteoforms place greater demands on the development of site-selective and accurate PTM installation methods. For large molecular weight proteoforms (over 100 kDa), scalability and cost would be

considerable aspects. A collaborative approach involving chemically synthesized protein domains bearing PTMs combined with expressed protein domains without PTMs could be a strategy to address this. The quality control and verification of the synthesized proteoforms will also require attention, utilizing interdisciplinary characterization techniques. As a risk-mitigating measure, a non-modified sample should be synthesized first and compared with the recombinant counterpart; this practice could validate the synthetic routine. The future research directions are anticipated to focus on the development of highly efficient and scar-less transpeptidative ligation of protein domains under non-denaturing conditions. This approach is feasible, as most protein structural domains are less than 300 amino acids in length and can be efficiently synthesized using current ligation and refolding strategies."

Additional Questions:
Quality of experimental data, technical rigor: High
Significance to chemistry researchers in this and related fields: Top 5%
Broad interest to other researchers: Top 5%
Novelty: High
Is this research study suitable for media coverage or a First Reactions (a News & Views piece in the journal)?: No

We thank the reviewer again for the comments and valuable suggestions that are very helpful for improving the quality of our manuscript.

Reviewer: 3

Recommendation: Publish in ACS Central Science after minor revisions noted.
Response: We thank the reviewer for supporting the publication of our manuscript.

Comments:
This paper effectively addresses a critical challenge in human health research: understanding the function of complex human proteoforms. The authors outline the key chemical advancements needed for efficient and precise synthesis of these molecules, including novel peptide ligation strategies for PTM incorporation, temporary structural support for folding, and efficient domain ligation methods. They further explore the applications of chemically synthesized proteoforms in dissecting protein-protein interaction networks, elucidating structures and functions of proteoform complexes, and developing drugs targeting specific proteoforms. By highlighting the need for new chemistries, the paper lays out a roadmap for future advancements in this transformative field.
Response: We thank the reviewer for the positive comments.

Here are some additional points the authors might consider including in this paper:
1. Briefly mentioning the vast number of human proteoforms due to PTM combinations. This emphasizes the need for efficient and scalable chemical synthesis methods.
Response: We thank the reviewer for the suggestion and have added this to our revised manuscript.

*"From the same genetic template, various factors including DNA genetic variation, RNA alternative splicing, and especially the diverse types and combinations of protein posttranslational modifications (PTMs) can lead to the generation of a wide range of structurally distinct proteins (**Figure 1**), known as proteoforms"*

2. Briefly discussing the limitations of current methods for studying proteoforms (e.g., recombinant expression) and how chemical synthesis offers advantages.
Response: We apologize that this section was not more prominently featured in the previous version of the manuscript, and we have revised the text as follows:

"*Traditional biological approaches like recombinant expression or cellular extraction often struggle to provide proteoform samples with the structurally precise and defined PTMs necessary for research. The inherent heterogeneity or limitations of these methods make it challenging to obtain the highly pure, homogeneous proteoforms required for in-depth studies. In contrast, chemical protein synthesis offers a potent de novo construction approach. By incorporating desired PTM-carrying building blocks, and then performing precise peptide or protein domain ligation and refolding, this methodology provides access to well-defined proteoform samples that are otherwise difficult to obtain through traditional biological techniques. This approach furnishes a valuable tool for advancing biomedicine research.*"

3. Addressing some of the main challenges in chemically synthesized proteoforms, such as the complexity of PTM incorporation, scalability and cost considerations, verification and quality control methods, and future research directions in this field.
Response: We thank the review for the suggestion and have discussed these challenges in chemically synthesized proteoforms in the Outlook section.

"However, chemical protein synthesis does face some inherent limitations. The most prominent challenge is the scalability and efficiency of synthesis, particularly for large, complex protein domains. The step-wise nature of chemical ligation and the potential for side reactions or incomplete modifications can lead to lower yields and purity as the target protein size increases. Additionally, the proper refolding of larger, multi-domain proteins

remains a technical hurdle. To address these limitations, future research should explore strategies such as automated synthesis platforms, computational design of optimal ligation schemes, and the development of new building blocks and catalysts to streamline PTM incorporation. Advancements in analytical techniques for rapid characterization of synthetic proteoforms will also be crucial to validate the accuracy and fidelity of the chemical synthesis approach.

There are still many challenges in the pursuit of chemical synthesis of the entire human proteome. The diverse PTMs on human proteoforms place greater demands on the development of site-selective and accurate PTM installation methods. For large molecular weight proteoforms (over 100 kDa), scalability and cost would be considerable aspects. A collaborative approach involving chemically synthesized protein domains bearing PTMs combined with expressed protein domains without PTMs could be a strategy to address this. The quality control and verification of the synthesized proteoforms will also require attention, utilizing interdisciplinary characterization techniques. As a risk-mitigating measure, a non-modified sample should be synthesized first and compared with the recombinant counterpart; this practice could validate the synthetic routine. The future research directions are anticipated to focus on the development of highly efficient and scar-less transpeptidative ligation of protein domains under non-denaturing conditions. This approach is feasible, as most protein structural domains are less than 300 amino acids in length and can be efficiently synthesized using current ligation and refolding strategies. "

Therefore, I recommend a minor revision for this paper to be considered for acceptance.
Response: We thank the reviewer again for supporting the publication of our manuscript.

Additional Questions:
Quality of experimental data, technical rigor: Top 5%
Significance to chemistry researchers in this and related fields: Top 1%
Broad interest to other researchers: Top 5%
Novelty: Top 5%
Is this research study suitable for media coverage or a First Reactions (a News & Views piece in the journal)?: Yes

We thank the reviewers again for the comments and valuable suggestions that are very helpful for improving the quality of our manuscript.
We wish that the revised manuscript could be accepted for publication in *ACS Cent. Sci.* Your kind consideration is highly appreciated.

With my best wishes,

Lei Liu, Ph.D.
